# Long-Term Potentiation and Excitability in the Hippocampus Are Modulated Differently by θ Rhythm

**DOI:** 10.1523/ENEURO.0236-18.2018

**Published:** 2018-11-22

**Authors:** Clayton S. H. Law, L. Stan Leung

**Affiliations:** 1 Department of Physiology and Pharmacology, The University of Western Ontario, London, Ontario N6A 5C1, Canada,; 2 Graduate Program in Neuroscience, The University of Western Ontario, London, Ontario N6A 5C1, Canada

**Keywords:** θ rhythm, long-term potentiation, basal dendrites, apical dendrites, population spike

## Abstract

Oscillations in the brain facilitate neural processing and cognitive functions. This study investigated the dependence of long-term potentiation (LTP), a neural correlate of memory, on the phase of the hippocampal θ rhythm, a prominent brain oscillation. Multichannel field potentials and current source-sinks were analyzed in hippocampal CA1 of adult male rats under urethane anesthesia. A single burst (five pulses at 200 Hz) stimulation of stratum oriens (OR) induced LTP of the basal dendritic excitatory sink (ES), which was maximal when the burst was delivered at ∼340° and ∼160° of the distal dendritic θ rhythm. Apical dendritic sink evoked by stratum radiatum (RAD) stimulation also showed biphasic maxima at ∼30° and ∼210° of the distal dendritic θ rhythm, about 50° phase delay to basal dendritic LTP. By contrast, maximal population spike (PS) excitability, following single-pulse excitation of the basal or mid-apical dendrites, occurred at a θ phase of ∼140°, and maximal basal dendritic ES occurred at ∼20°; γ (30–57 Hz) activity recorded in CA1 RAD had maximal power at ∼300° of the distal dendritic θ rhythm, different from the phases of maximal LTP. LTP induced during the rising θ phase was NMDA receptor sensitive. It is suggested that the θ phase modulation of CA1 PS excitability is mainly provided by θ-rhythmic proximal inhibition, while dendritic LTP is also modulated by dendritic inhibition and excitation, specific to basal and apical dendrites. In summary, basal and apical dendritic synaptic plasticity and spike excitability are facilitated at different θ phases in a compartmental fashion.

## Visual Abstract

**Figure:**
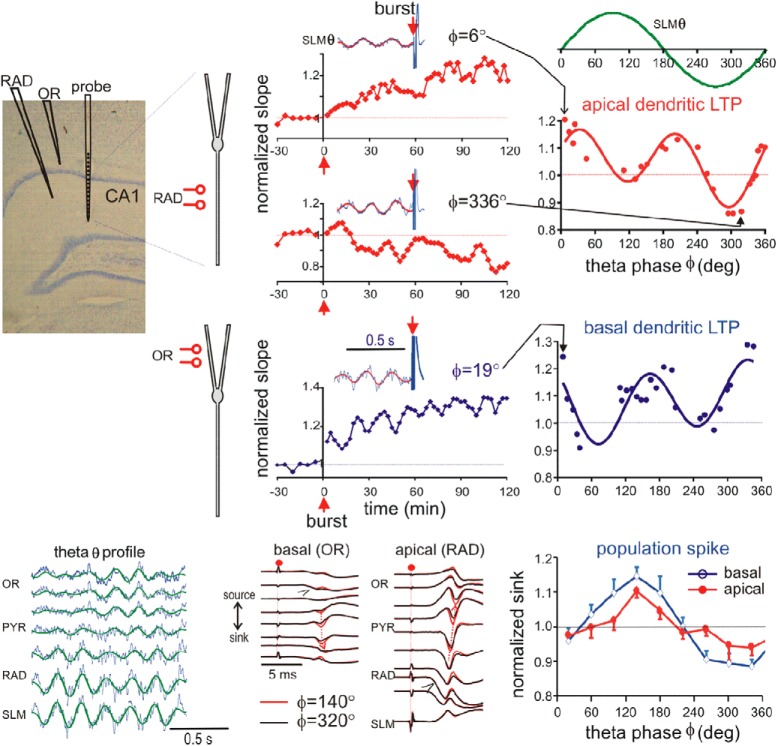

## Significance Statement

How the hippocampal θ rhythm modulates synaptic plasticity and transmission is not completely known. Here, we showed, for the first time, phase modulation of synaptic plasticity and spike excitability across a complete θ cycle. Using a single burst stimulation to probe long-term potentiation (LTP) in hippocampal CA1, we observed two LTP peaks and a single spike excitability peak in a single θ cycle (360°). Basal dendritic LTP led apical dendritic LTP by ∼50°, and LTP peaks occurred during both low and high spike excitability. This suggests a brain oscillation can optimally process output (spikes) and synaptic plasticity of different dendritic compartments at different phases, possibly resulting from rhythmic somatic and dendritic inhibition and excitation in the network.

## Introduction

A brain oscillation is suggested to facilitate storage and retrieval of information in neural circuits (Sejnowski and Paulsen, 2006), but the mechanisms are not clear. The present study attempts to elucidate the neural processes whereby a hippocampal θ rhythm facilitates synaptic plasticity. A hippocampal θ rhythm accompanies an active brain in animals (Vanderwolf, 1969; Bland and Colom, 1993; Leung, 1998; Buzsáki, 2002) and humans (Tesche and Karhu, 2000; Kahana et al., 2001). θ Rhythm is suggested to facilitate spatial navigation and memory (O'Keefe and Recce, 1993; Dragoi and Buzsáki, 2006). Abolition of the hippocampal θ rhythm disrupts hippocampus-dependent memory (Winson, 1978) and its consolidation during rapid-eye-movement sleep (Boyce et al., 2016).

How θ rhythm promotes memory encoding is not known, but one mechanism is to facilitate long-term potentiation (LTP), a cellular correlate of memory (Martin et al., 2000; Bliss et al., 2007). Repeated bursts at 5–10 Hz (θ frequency) were optimal for induction of hippocampal LTP (Larson and Lynch, 1986; Capocchi et al., 1992). Hippocampal LTP was induced when a brief high-frequency burst stimulation (five pulses at 200/400 Hz) was delivered to the positive, but not the negative, phase of θ rhythm. This was shown in the dentate gyrus following medial perforant path stimulation (Pavlides et al., 1988; Orr et al., 2001). In hippocampal CA1, apical dendritic synapses in stratum radiatum (RAD) showed LTP when three burst stimulations were given during the positive phase of RAD θ rhythm in urethane-anesthetized rats (Hölscher et al., 1997); no LTP or long-term depression (LTD) was found when bursts were delivered to the θ negative phase. Similarly, apical dendritic LTP was induced in behaving rats when three bursts occurred at the positive phase, but LTD was induced when the bursts occurred at the negative phase of RAD θ (Hyman et al., 2003). LTP and depotentiation *in vitro* were induced at positive and negative phases, respectively, of the carbachol-induced θ oscillation (Huerta and Lisman, 1995). Despite the above studies, the relation of LTP with phase across a complete θ cycle is not known.

θ Frequency modulation of synaptic responses and plasticity likely results from θ-rhythmic inhibitory and excitatory conductance changes in the somata and dendrites of CA1 pyramidal cell (Leung and Yim, 1986; Ylinen et al., 1995; Kamondi et al., 1998). Soma-directing and dendrite-directing interneurons, firing at different θ phases, provide phase-dependent inhibition of different pyramidal cell compartments (Ylinen et al., 1995; Kamondi et al., 1998; Klausberger and Somogyi, 2008). θ-Rhythmic excitation of the proximal and distal dendrites of CA1 pyramidal cells comes from CA3 and entorhinal cortex (Kocsis et al., 1999; Mizuseki et al., 2009). Septohippocampal neurons in the medial septum may ultimately modulate the θ frequency inputs (Stumpf, 1965; Stewart and Fox, 1990; Freund and Buzsáki, 1996; Borhegyi et al., 2004; Vandecasteele et al., 2014; Robinson et al., 2016). As a consequence of the somatic-inhibitory and dendritic-excitatory modulation, θ field potentials in CA1 showed a gradual phase shift in the apical dendritic layers (Winson, 1974; Buzsáki et al., 1983; Leung, 1984; Brankack et al., 1993; Buzsáki, 2002). Because of overlapping dipole fields in CA1, whether LTP occurs at the positive phase of a local θ rhythm has no clear neurophysiological meaning.

The main goal of the present study was to quantitate the dependence of LTP in CA1 on a complete range of θ phase (0–360°), instead of focusing on positive and negative θ phases as in previous studies. Instead of using a θ phase reference in RAD, where a rapid change of θ phase with depth occurs, a depth profile of θ field potentials was recorded, and a more stable and reliable θ reference in stratum lacunosum moleculare (SLM) was used. A single burst, rather than multiple bursts, was used to induce synaptic plasticity, so that the θ phase at the onset of one burst can be accurately estimated. Synaptic plasticity was studied at both the basal and apical dendritic synapses of CA1 pyramidal cells in urethane-anesthetized rats, using current-source density (CSD) analysis to localize the active current sink at the basal or apical dendrites. In addition, the θ phase dependence of LTP was compared to that of the evoked population spike (PS), following basal or apical dendritic excitation, and of γ and ripples activity in the local field potentials (LFPs). We reported that the biphasic LTP was modulated by a θ 2nd harmonic, while the single peak of spike excitability or γ LFP power was modulated by the θ fundamental (1st harmonic).

## Materials and Methods

### Surgery and electrode implantation

Adult male Long-Evans rats, weighing 230–350 g, were used (Charles River Laboratories). Data were derived from 90 rats. Rats were housed in standard cages in a temperature-regulated environment in a 12/12 h light/dark cycle commencing at 7 h, with *ad libitum* access to food and water. Experiments were conducted during 9–20 h.

Rats were anesthetized using urethane (1.5 g/kg, i.p.), with atropine methyl nitrate (5 mg/kg, i.p.) to reduce airway secretion, and placed in a stereotaxic frame. The rat’s rectal temperature was maintained at 36.5–37°C via feedback heating. Monopolar stimulating electrodes (127-µm diameter stainless steel wire, Teflon-coated except at cut ends) were lowered into RAD at P3.2, L3.2, ventral from skull surface (V) ∼3.0 (all units in mm) and stratum oriens (OR) at P3.2, L2.2, V ∼2.5, with bregma and λ on a horizontal plane (Paxinos and Watson, 1998; Fung et al., 2016). Two screws were secured in the skull over the cerebellum and frontal cortex to serve as the stimulus anode and recording ground. Cathodal pulses were delivered to the RAD and OR stimulating electrodes, and a stimulating electrode was optimized to evoke apical or basal dendritic responses from CA1 pyramidal cells. A silicon probe with 16 recording sites spaced 50 µm apart on a vertical shank (A1x16-5mm-100-177; NeuroNexus) was lowered into CA1 at P3.8, L2.0, V ∼3.0, to record evoked population EPSPs (pEPSPs; Kloosterman et al., 2001; Fung et al., 2016). Signals from the silicon probe were amplified by a headstage (Tucker-Davis Technologies; TDT) and fed into a Medusa preamplifier and digital processors (RA16 Base Station). Signals were digitized at 6.1 or 24.4 kHz by TDT real-time processors and custom-made software. Stimulus pulses (0.2-ms duration) were delivered through a photo-isolated stimulation unit (PSIU6, AstroMed) driven by a Master 8 pulse generator (AMPI).

CSD analysis was used to locate the excitatory sink (ES) and associated source. One dimensional CSD (z, t) was calculated by a second-order differencing formula (Leung, 2010):

CSD (z, t) = σ [2 Φ(z, t) - Φ(z + nΔz, t)- Φ(z - nΔz, t)]/(nΔz)^2^ where Φ(z, t) is the potential at depth z and time t, Δz is the spacing (50 µm) between adjacent electrodes on the 16-channel probe, and *n* = 2 was used for spatial smoothing. Conductivity σ was assumed to be constant and CSDs were reported in units of V/mm^2^.

Baseline evoked responses were monitored at 1.5–2× threshold intensity at a sampling rate of 24.4 kHz. Four single sweeps, repeating at 0.1 Hz, and the average evoked potential (AEP) were digitized and stored every 2.5–5 min. After a stable baseline was obtained for 30 min, a single burst consisting of five stimulus pulses at 200 Hz and 3–3.5× threshold intensity was delivered to either the RAD or OR stimulating electrode to induce synaptic plasticity. A single burst at 100–200 Hz was reported to induce Schaffer-collateral mediated LTP in CA1 *in vivo* (Hölscher et al., 1997) or *in vitro* (Remy and Spruston, 2007). A short burst at 200 Hz simulates the natural firing of single hippocampal pyramidal cells (Ranck, 1973). Previous studies used a stimulation intensity that elicited 90% of the maximum pEPSP (Hölscher et al., 1997; Hyman et al., 2003), which ranged from 4–6× threshold intensity in our preparations.

Spontaneous LFPs were recorded from 16 channels before, during, and after the burst stimulation. If θ rhythm was not spontaneously present, it was induced by pinching the base of the rat’s tail. The stimulus burst can be targeted at different time delays from the peak (or trough) of the θ rhythm at one electrode, by means of RPvdsEx software (TDT). However, the exact θ phase at the onset of the burst stimulation was analyzed from the recorded LFPs (below).

Potentiation (LTP or lack of) was analyzed by one of four methods. (1) ES maximal slope, with the CSD derived from the AEP; the maximal slope is defined as the largest slope over 1-ms time interval during the rising phase of the ES, at the electrode (channel) in OR (or radiatum) which had the largest response. (2) ES maximal slopes derived from single sweeps of evoked potentials; giving 24–48 values in a 30-min block for assessing the variability (SD) of the response. Other alternative measures derived from the AEP were: (3) the sink amplitude at approximately one third of the rise to peak, typically 2- to 3**-**ms latency from onset, and (4) the sum of sinks at the above time instant. The alternative measures (3) and (4) gave similar results as the ES maximal slope (Fung et al., 2016) and will not be further reported. Stability during baseline was estimated by the SEM divided by the mean, and this ratio had to be <0.05 for 30 min before burst stimulation. After burst stimulation, responses were taken at 5**-**min intervals, from 5 to 120 min; some rats were recorded at 2.5**-**min intervals starting at 2.5 min post-burst. For each experiment, all measures were normalized by the average measure during baseline before burst delivery. CSD depth profiles were constructed at a single instant of time, and experiments in which the peak source-sink pattern shifted 50 μm or more were discarded.

A frequency distribution of the single-sweep slope amplitudes was constructed for a 30-min block of baseline (*n* = 24 sweeps), and post-burst responses (*n* = 24 or 48 sweeps). Single-sweep slope amplitudes were normalized by the baseline average, and plotted as the fraction of responses in different bins away from the mean, with one bin width equal to the SD of the distribution during baseline (SD_base_). In LTP experiments, an evoked potential trace was 167 ms long, which precluded estimate of δ and θ activity or brain state from the LFP. As reported elsewhere (Leung and Péloquin, 2010) and in the following Results, the apical dendritic ES slope (following RAD stimulation) was higher by ∼20%, and the basal dendritic ES slope (following OR stimulation) by ∼10%, during non-θ as compared to θ LFP. Since the LFP in urethane anesthetized rats cycled between θ and non-θ states in a period of ∼11 min (Clement et al., 2008), averaging LTP over a period of 30 min is expected to average across both θ and non-θ states.

### Dependence of LTP on NMDA receptor antagonist

To test whether the burst-stimulation induced synaptic plasticity was dependent on NMDA receptors, NMDA receptor antagonist 3-(2-carboxypiperazin-4-yl)propyl-1-phosphonic acid (CPP) 1 mM dissolved in 0.9% physiologic saline, or 0.9% saline, was loaded into a 1-mm-diameter micropipette broken to make a tip of ∼2 μm in diameter. The micropipette was lowered into CA1 at P3.5, L1.8, V ∼2.5 with an angle of ∼15° from vertical to reach <1 mm of the silicon probe placed in CA1. CPP was allowed to diffuse into surrounding areas for 60 min before taking baseline measurements. The local delivery of CPP avoids possible systemic disruption of θ, e.g., through the medial septum (Leung and Shen, 2004). A single burst was delivered to either OR or RAD during the rising phase of SLM θ, attempting to induce LTP in the presence of CPP or saline (control). Burst induced sink at the dendritic site was analyzed by sink integration of an early phase (0–20 ms after 1st pulse of the burst), and late phase (5 ms after the 5th pulse to zero crossing of the sink), and the duration of the sink.

### Evoked PS and ES during θ versus non-θ states

A single high-intensity electrical stimulus was delivered to either OR or RAD to evoke a PS, at ∼1.5× the PS threshold. Stimulus pulses were delivered at 20-s intervals and traces were sampled at 12.2 kHz, allowing at least 1 s of LFP before the stimulus. The spontaneous background LFP activity cycled between periods of θ and non-θ activity, and LFP activity preceding the stimulus was determined to be θ or non-θ based on power spectral analysis (Leung et al., 1982). If a power peak occurred at a frequency between 3 and 6 Hz, it was considered θ activity. Non-θ slow activity had peak power occurring at 1–2.9 Hz. The θ to low-δ (1–2.9 Hz) power ratio was also calculated as the ratio of the sum of θ (3–6 Hz) power to the sum of low-δ power. PS amplitude was measured from the CSD time trace at the pyramidal cell layer (PYR) as the amplitude of a vertical line drawn between the negative sink peak and a tangent line drawn between the two positive peaks before and after the negative sink. ES amplitude was measured as the maximal rising slope (over 1 ms) of a spatially maximal dendritic sink. Voltage of the PS (in mV) at PYR, and the slope of the pEPSP at the dendritic layer (in mV/ms), were also measured in potential records.

At the end of recordings, each stimulating electrode was marked by passing 0.5-mA current of 0.5-s duration, repeated three times. The rat was then intracardially perfused with 50 ml of saline followed by 50 ml of 4% formaldehyde solution. The brain was removed and placed in a 4% formaldehyde solution for >24 h before sectioning by a freezing microtome. The stimulus electrode locations and the track of the recording probe were identified in 40-µm-thick, coronal brain sections stained with thionin.

The depths of OR, stratum pyramidale (PYR), RAD, and SLM were determined by electrophysiological criteria. RAD stimulation evoked apical dendritic sink in RAD, which was surrounded by a major source in PYR and a minor source in SLM (Kloosterman et al., 2001). The latter sources were used to identify PYR and SLM, which was separated by 8.05 ± 0.07 channels (*n* = 50) or 400 μm. In addition, OR stimulation evoked maximal sink in the same layer, accompanied by a source in PYR. Electrophysiological determination of CA1 layers was verified in previous studies by making small lesion through a recording channel (Townsend et al., 2002). In the present study, the track of the silicon probe in CA1 was confirmed in thionin-stained sections.

### Experimental design and statistical analysis

**Figure 1.**
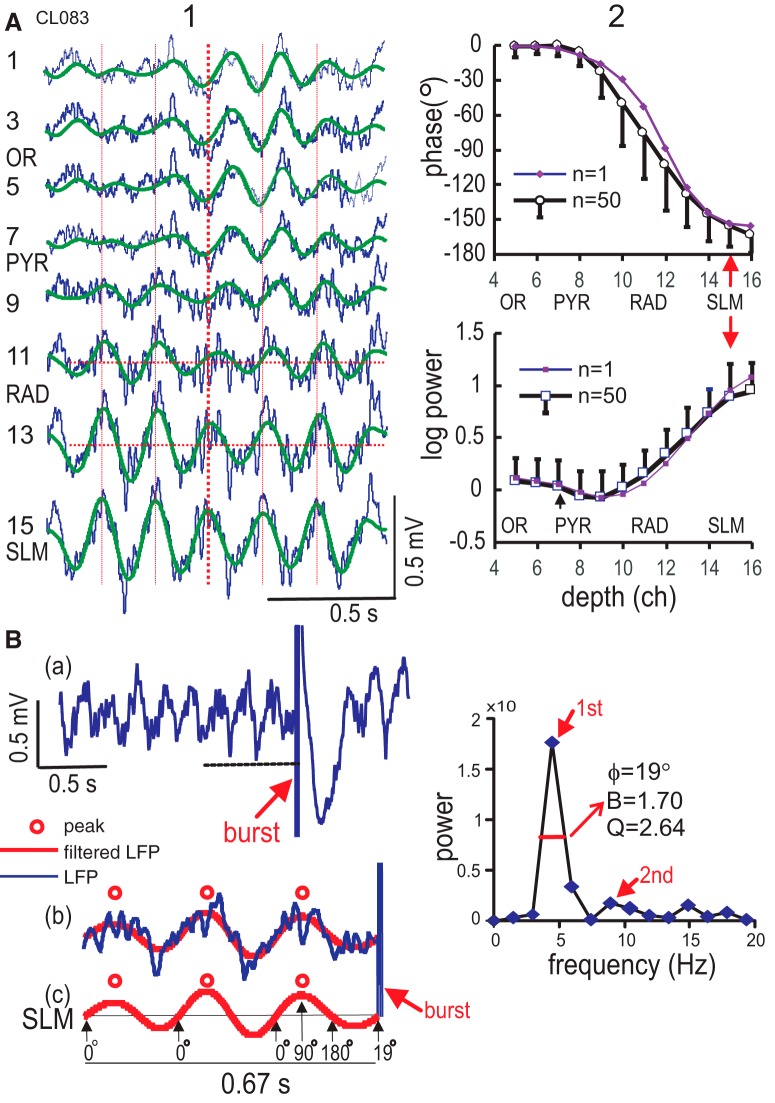
Phase profile of θ rhythm and phase reference at the distal dendritic layer. ***A***, Laminar profile of θ rhythm recorded in selected odd channels of a 16-channel array with raw LFPs (thin trace) superimposed on 0–8 Hz digitally filtered signal (thick trace). θ Recorded in different layers: OR, PYR, RAD, and SLM. θ Phase shift was large in RAD, as could be seen from θ peak alignment with the vertical line. Column 2, Phase (top) and logarithmic power (bottom) profiles of the spontaneous θ rhythm (average of 10 segments of 1.34 s) in a single rat (CL083; *n* = 1), or a group of rats (*n* = 50), with error bars indicating one SD. The group data aligned SLM as ch15 in all rats; SLM θ showed near-maximal power and minimal phase SD. ***B***, Estimate of θ phase at onset of burst stimulation. Column 1. Trace (a), LFP recorded at SLM shows the onset of the stimulus burst (arrow; five pulses at 200 Hz) at the rising phase of the θ rhythm. Trace (b) shows an expanded trace (horizontal line in a) of the LFP (thin trace), and digitally filtered (0–8 Hz) signal (thick trace) with peaks indicated by open circles. Trace (c) shows the filtered LFP at SLM, labeled by phase of a sine wave (0° onset, 90° peak and 270° trough). Column 2, Power spectrum, of 0.67-s LFP immediately before the burst, shows peaks at the θ 1st (4.47Hz) and 2nd harmonic (8.95 Hz), the half-peak bandwidth (B), resonance (Q), and phase estimate (ϕ = 19°) of burst onset with reference to SLM θ.

Custom-made MATLAB programs (MathWorks) were used to determine whether the burst stimulation occurred during θ activity, and the θ phase at the onset of the burst. A segment of LFP at SLM (typically around channel 15) immediately preceding the onset of the burst, 4096 points sampled at 6.103 kHz (0.67-s duration), was used for power spectral analysis using Fast Fourier Transform (FFT). The half-peak bandwidth (B; in Hz) was estimated by linear extrapolation around the peak power, and resonance (Q), or quality of the peak, was estimated by Q = P/B, where P was the peak frequency (frequency resolution 1.49 Hz). Acceptable criteria for θ in the LFP segment were a peak frequency within a frequency range of 2.98–6 Hz in the power spectrum, and a resonance Q ≥ 1 (Fig. 1*B2*). The preferred “time-peak” method for estimating the θ phase at the onset of the burst used 0- to 8-Hz digital filtering of the LFP immediately before the burst (forward FFT, removes >8-Hz frequencies, and then inverse FFT, with no phase-shift; Fig. 1*B*). The θ frequency was estimated by the inverse of the interval between the last two peaks, and the θ phase was estimated by (time from last LFP peak to onset of first pulse of the burst)/(interval between two filtered LFP peaks) * 360° plus 90°. The present study used the SLM θ as the reference signal, and phase is referred to a sine wave that starts at 0° and peaks at 90° (Fig. 1*B1*). In each rat, a depth profile of θ phase was estimated from the spontaneous LFPs (4–10 segments of 8192 points) selected for θ (Fig. 1*A1*,*A2*). Some studies used the PYR θ as the reference signal, which showed an amplitude <50% of the SLM θ, as seen in the depth profile of the θ power (Fig. 1*A2*).

Alternatively, a “FFT method” estimated the θ phase at the onset of the burst (using 4096–8192 points). At the SLM channel, the θ phase was estimated by cross-spectral analysis with a pure sine wave constructed from a single dominant digital θ frequency. The FFT method approximates a single θ frequency across the pre-burst time segment, instead of using θ frequency determined from the last two θ peaks in the time-peak method. The phase accuracy of the FFT method was affected by peak dispersion (i.e., a broad θ peak) and by the low digital frequency resolution (1.49–0.74 Hz). In general, for the present study, phase estimates from the FFT method gave similar results as the time-peak method.

Cross-frequency coupling of different LFP bands to θ rhythm (Tort et al., 2009; Belluscio et al., 2012) was estimated. LFP frequency bands were categorized as low γ (30–57 Hz), high γ (63–100 Hz), low ripples (100–250 Hz), and high ripples (250–400 Hz). LFP was estimated from signals recorded at a single electrode. The LFP was FFT filtered to obtain a particular frequency band, inverse FFT filtered, and Hilbert transformed to derive the envelope. Cross spectral (coherence and phase) analysis was performed for the envelope and the raw (unfiltered) signal. Cross-frequency phase estimate of a rat was included when the coherence showed a peak of >0.005 at a θ frequency (2.98–6 Hz).

Potentiation as a function in time was determined from normalized CSD slopes of a group of rats. Comparison between potentiation (or other measure) of two groups used a two-factor (group × time) repeated measures ANOVA. If a significant (*p* > 0.05) main or interaction effect was found, Newman–Keuls *post hoc* test was applied.

An average LTP versus θ phase profile was determined by a running average of three to four rats with phases within 60° of each other. The relation of running average LTP to phase was subjected to non-linear regression (nln function in MATLAB, which gave the same result as nonlinear regression in GraphPad Prism 7.0). The main curve fit used a sum of 1st and 2nd harmonic plus a constant, y = A0 + A1 sin (π/180 * (x + A2)) + B1 sin (π/90 * (x + B2)). A single sine wave at the 1st harmonic, y = A0 + A1 sin (π/180 * (x + A2)), or a single sine wave at the 2nd harmonic, y = A0 + B1 sin (π/90 * (x + B2)), was also used for curve fitting. For each equation, x = phase in degrees and y = running phase-averaged measure (LTP or other); parameter A0 is a constant near unity; A1 and A2 are amplitude and phase shift (in degrees) of the 1st harmonic, respectively; B1 and B2 are amplitude and phase shift of the 2nd harmonic, respectively. The *F* statistic was used to give the probability (*p*) of random association between x and y. Custom made software was also used to permute random pairing of x and y values (at least 200 times), to estimate the probability that the goodness of fit parameter *R* ^2^ is larger than a particular value; the latter probability is almost identical to that given by the *F* statistic.

For PS amplitude and ES slope/pEPSP slope in relation to θ phase, the PS amplitude was grouped into 9 phase bins, each of 40° interval, starting at phase 0°. For each rat, measures within one phase bin were averaged, and the bin-average was normalized by the average across all phases. Group data were compared by one-way or two-way repeated-measures ANOVA, as appropriate; *p* < 0.05 was considered statistically significant. Comparison between two groups also used a Wilcoxon signed**-**rank test. The phase relation of the run-averaged PS, ES, or pEPSP slope was also subjected to a non-linear curve fit by a sum of 1st and 2nd harmonics plus constant, or by a 1st or 2nd harmonic plus constant, as described above. Values are presented as mean ± SEM, except when noted.

## Results

### θ Phase profile in CA1 and θ phase reference

To find an optimal reference θ signal, depth profiles of the θ phase and power were recorded. A representative profile of the spontaneous LFPs, with digitally filtered (0–8 Hz) signals (Fig. 1*A*) shows that at a fixed time (vertical line), a negative θ peak at OR decreased in amplitude in PYR, and slowly shifted to a positive peak in RAD and higher peak in SLM (Fig. 1*A*). The depth profile of θ power shows peaks at OR and SLM and a minimum at proximal RAD (Fig. 1*A2*, channel 9). The depth profile of θ phase shows ∼0° in OR and PYR, and then a gradual phase shift from PYR to SLM, reaching –153° at SLM (Fig. 1*A2*). For a group of rats used for LTP experiments (*n* = 50), the θ phase shift between SLM (aligned to channel 15) and OR (channel 4) was –155.7 ± 2.5° (mean ± SEM; Fig. 1*A2*). The lowest θ phase variability in the group was found at SLM, PYR, and OR (error bar was one SD in Fig. 1*A2*). θ At SLM showed phase stability and near-maximal amplitude/power within CA1, and thus it was selected to be the reference signal for subsequent analysis.

The θ phase at the onset of a stimulation burst (five pulses at 200 Hz) was determined from the SLM LFP recorded immediately before burst stimulation (Fig. 1*B1*). An LFP segment of 0.67-s duration was digitally filtered (0–8 Hz), and peaks of the low-pass filtered LFP were used to determine the θ phase at the onset of burst stimulation (Fig. 1*B1*; see Materials and Methods). The SLM θ is considered to be a sine wave that starts at 0°, peaks at 90°, and troughs at 270°, giving the phase estimate of burst onset to be 19° (Fig. 1*B*). The 0.67-s LFP segment was also subjected to power spectral analysis, resulting in a representative spectrum (Fig. 1*B2*) showing a 1st θ harmonic peak at 4.47 Hz, half-peak bandwidth (B) of 1.7 Hz, resonance (Q) of 2.64, and a 2nd θ harmonic peak at 8.94 Hz. The group mean ± SEM for the pre-burst LFP segments in all LTP experiments (*n* = 50) were B = 2.01 ± 0.08 Hz, Q = 2.24 ± 0.10, at a θ peak frequency of 4.17 ± 0.09 Hz.

### Basal dendritic LTP evoked by a single burst delivered to OR during θ

**Figure 2.**
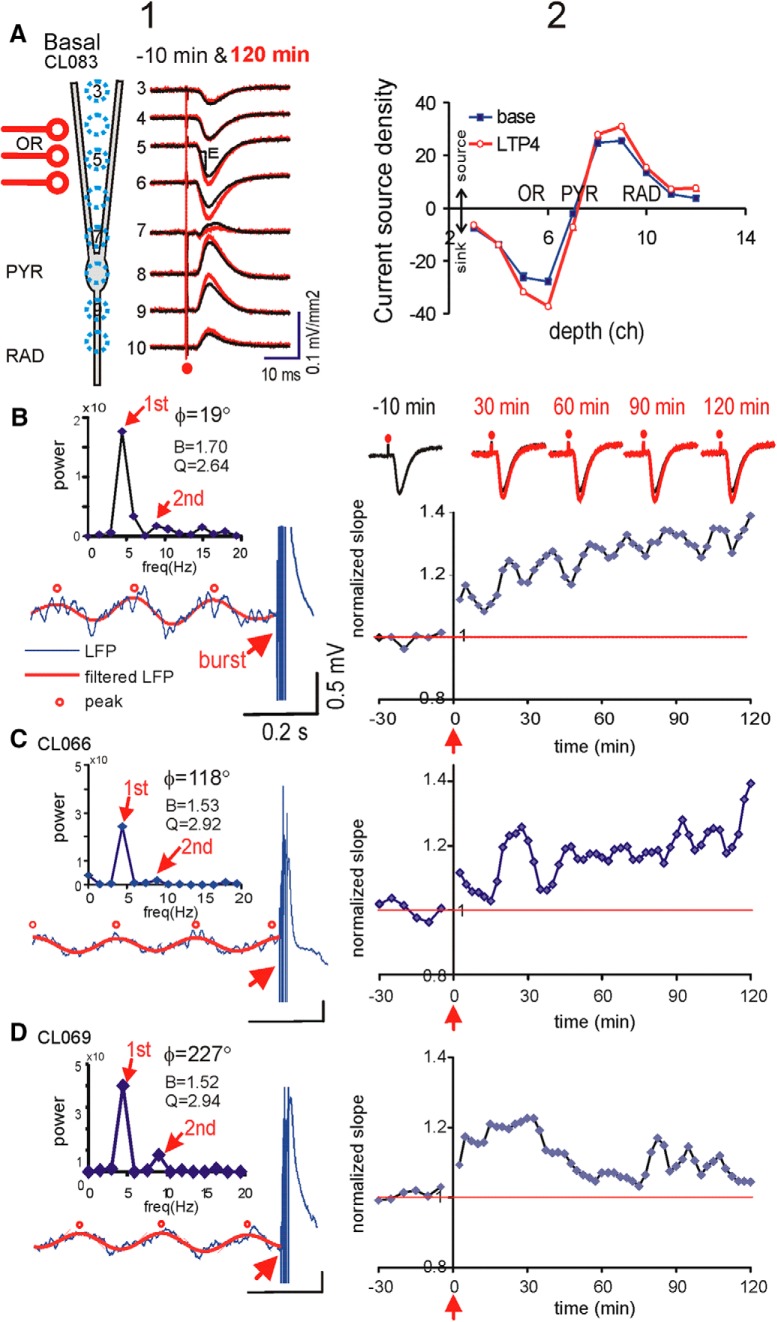
Burst stimulation during the rising phase of θ rhythm induced basal dendritic LTP. ***A***, Column 1. Schematic CA1 pyramidal cell is shown with a multichannel (channel 3–10) laminar profile of average CSD transients evoked by single-pulse stimulation of OR at 2× threshold intensity. Average CSDs during baseline (–10 min) and 120 min after burst stimulation are overlaid. OR stimulation evoked basal dendritic sinks, measured by the slope of the spatially maximal ES (E) at channel 5. Column 2, Depth profile of the CSDs, with a sink at OR and sources at PYR and RAD, was potentiated at 90–120 min (LTP4) compared to baseline. ***B***, column 1, Stimulus burst was delivered at the rising phase of SLM θ rhythm, estimated to be 19°; power spectrum (same as Fig. 1*B2*) shows peaks at 1st and 2nd θ harmonics, and the half-peak bandwidth (B) and resonance (Q). Right column 2 shows the time course of the normalized slope of the basal dendritic current sink that increased gradually after burst stimulation at time 0; top inset traces are average CSDs (12 sweep averages) during baseline (–10 min), and at 30, 60, 90, and 120 min after burst stimulation (thick trace), overlaid on the baseline (thin trace). ***C***, Same as ***B*** but for another rat with burst delivered at 118° θ phase, resulting in a slowly rising LTP. ***D***, Same as ***B*** but for another rat with burst delivered at 227° θ phase, resulting in larger early than late (>60 min) potentiation.

AEPs following single-pulse OR stimulation were analyzed to reveal a basal dendritic sink at OR, accompanied by current sources at PYR and RAD (Fig. 2*A1*,*A2*). The maximal slope of the basal dendritic sink, over a 1-ms interval, was used for quantitative assessment. After stable responses were recorded for 30 min (Fig. 2*B2*), a single stimulus burst consisting of five pulses at 200 Hz was delivered to OR. In a representative rat, the burst delivered at the rising phase of the SLM θ rhythm (Fig. 2*B1*) resulted in normalized sink slopes that increased immediately after burst stimulation (12% increase at 5 min), and then more gradually in the next 120 min (Fig. 2*B2*). Increase of basal dendritic response was confirmed by a spatial profile of CSDs, which showed that both the sink in OR and the source in PYR/proximal RAD were enhanced (Fig. 2*A1*,*A2*). In other rats, burst stimulation was delivered at different SLM θ phases. Similar but weaker LTP was induced by burst stimulation at ∼120° SLM phase (Fig. 2*C*). Burst stimulation at ∼230° (Fig. 2*D*) evoked higher LTP during early (0–30 min) than late (30–120 min) times. In the group, OR burst stimulation was delivered at approximately three times the threshold intensity (106.0 ± 3.9 μA, *n* = 26).

**Figure 3.**
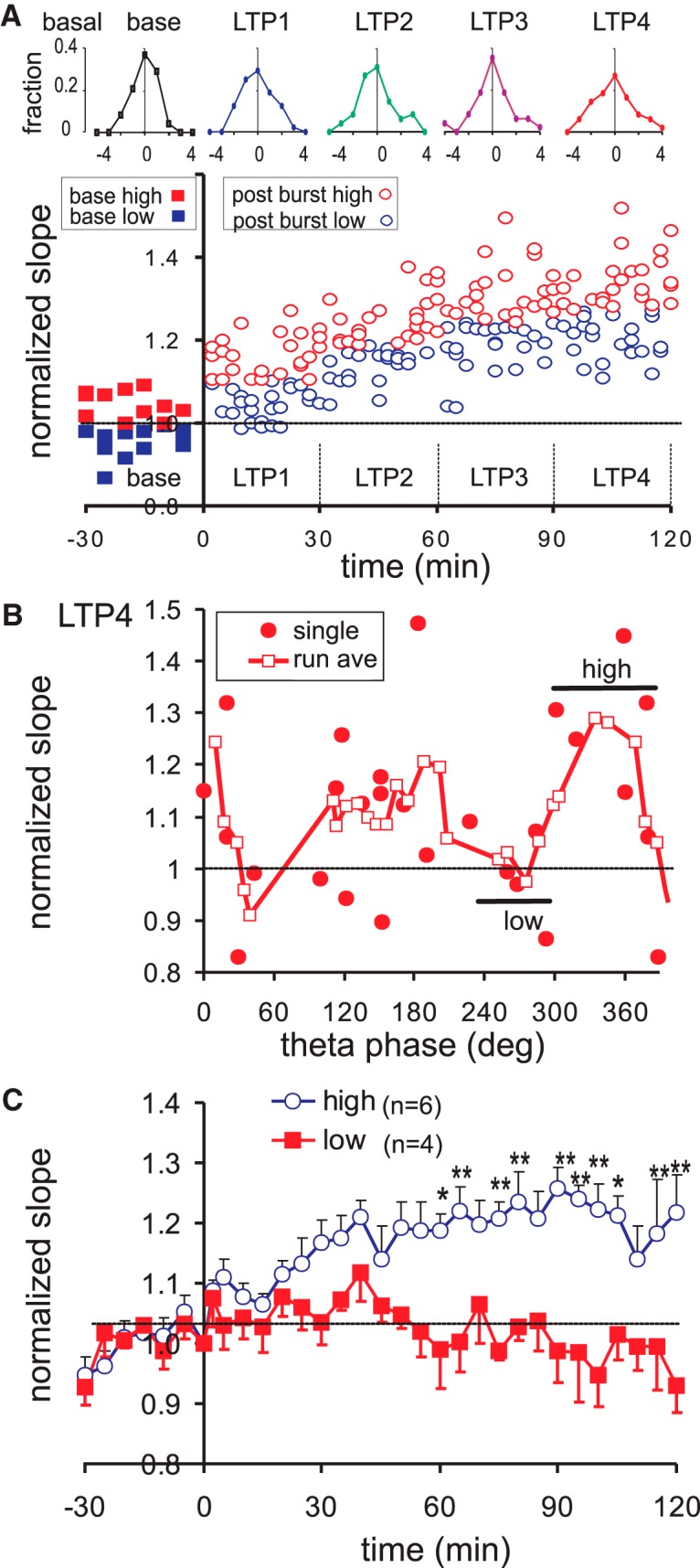
Variability of basal dendritic response in time, and relation of basal dendritic late LTP with SLM θ phase. ***A***, Time course of basal dendritic LTP illustrated by single-sweep response in a representative rat (same rat as in Fig. 2*B*); single sweep responses were sorted as above (high) or below (low) the median during a 30-min time period (LTP1–LTP4). Top row shows the frequency distribution at baseline and LTP1–LTP4; vertical axis labeled as fraction of occurrence in different bins away from the mean (horizontal axis), with 1 bin = SD during baseline. Frequency distribution did not change significantly with time periods. ***B***, Normalized slope of the basal dendritic ES of single rats (filled circles), and running phase-average of the normalized slope of three to four rats (run ave; open squares), are plotted with the SLM θ phase at burst onset; data points at θ phase 360–390° are the same as those at 0–30°. ***C***, Mean ± SEM of the normalized ES slope was plotted for the high and low groups indicated in ***B***. Two-way repeated measures ANOVA showed a significant group effect *F*_(1,8)_ = 12.8, *p* < 0.01, with significant Newman–Keuls *post hoc* differences indicated by *p < 0.05; **p < 0.01.

The variability of the response was evaluated by the ES slopes, normalized by the baseline average, following each single-pulse stimulation (Fig. 3*A*). Frequency distribution of the normalized sink slopes, plotted in units of SD_base_ (Fig. 3*A*, top row) shows that the distribution was similar among different 30-min blocks of time, including baseline and four post-burst periods called LTP1 (0–30 min post-burst), LTP2 (30–60 min), LTP3 (60–90 min), and LTP4 (90–120 min). In the basal dendritic LTP group, SD_base_ was 0.086 ± 0.005 (*n* = 26 rats; mean =1), the SDs of the five 30-min blocks (baseline, LTP1–LTP4) were not found to be statistically different [*F*_(4100)_ = 1.03, *p* > 0.3, one-way repeated measures ANOVA]. The stability of the SD suggests that brain state variability among 30-min blocks was low. If the single sweep sink slopes were divided by the median into upper and lower groups for each 30-min block (Fig. 3*A*), the potentiation for the upper and lower groups was similar in time in the example (Fig. 3*A*) and in the group (data not shown).

A running average of basal dendritic LTP with θ phase was constructed. Each running average consisted of three to four points within 60° phase range (one point per rat). As illustrated for LTP4, its running average displays two maxima with θ phase at ∼160° and ∼340°, each preceded by a minimum (Fig. 3*B*). Group average of the time course of potentiation of the “high” group (at ∼340° θ phase) shows a gradual increasing potentiation, while group average of the minimum at ∼270° θ phase (“low” group) did not show LTP (Fig. 3*C*). There was no difference in the potentiation between low and high groups at 2.5 min after bursting, but the group differences were statistically significant at >60 min post-burst (Fig. 3*C*, * and **).

### Apical dendritic LTP evoked by a single burst delivered to RAD during θ

**Figure 4.**
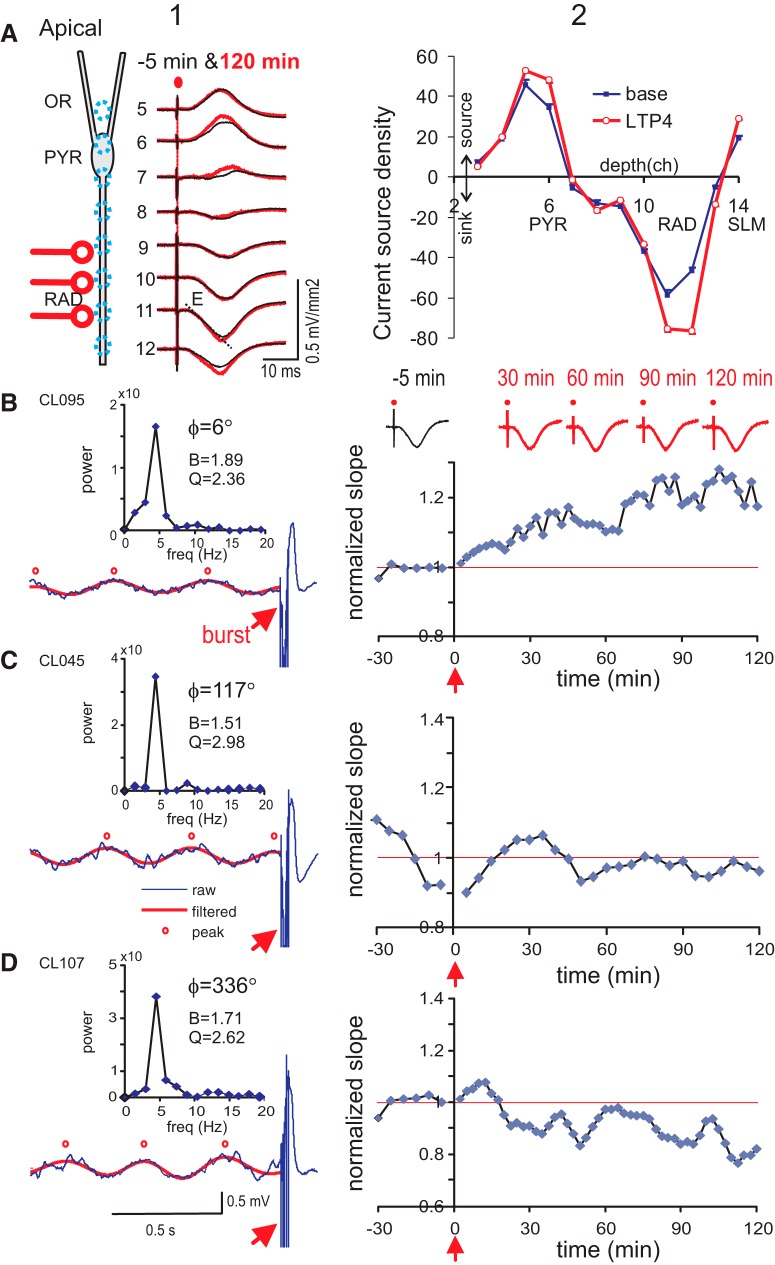
Burst stimulation during the rising phase of θ rhythm induced apical dendritic LTP. ***A***, left column 1, Laminar profile of CSD evoked by single-pulse, 2× threshold RAD stimulation in a representative rat (CL095) shows ES at RAD accompanied by major and minor sources at PYR and SLM, respectively. Average CSDs are overlaid for baseline (–5 min) and 120 min after burst stimulation; the burst landed on the rising phase of the SLM θ. Right column 2, CSD depth profile shows an increased apical dendritic sink (and accompanying source) at 90–120 min post-burst (LTP4; 48 sweeps averaged) as compared to baseline (24 sweeps averaged). ***B***, left column 1, Stimulus burst was delivered at the rising phase (∼6°) of SLM θ; power spectrum shows peaks at the 1st (4.47 Hz) and 2nd θ harmonics, and half-peak bandwidth (B) and resonance (Q). Right column 2 shows normalized slope of the apical dendritic current sink. Top traces are CSD averages during baseline (–5 min), and at different times post-burst. ***C***, Same as ***B*** for another rat with no significant potentiation induced by a burst delivered at 117° of the SLM θ. ***D***, Same as ***C*** for another rat with a burst delivered at 336° SLM θ phase, resulting in apparent late long-term depression.

Single-pulse RAD stimulation evoked apical dendritic sinks at the same layer, accompanied by current sources at PYR and SLM (Fig. 4*A1*,*A2*). After stable responses were recorded, a burst was delivered near 0° phase of the SLM θ (Fig. 4*B1*), RAD-evoked apical dendritic sink showed a normalized slope response that increased slowly with time (Fig. 4*B2*). After the burst, the apical dendritic sink at RAD, and the accompanying sources at PYR and SLM, were all increased compared to baseline (Fig. 4*A1*,*A2*). Burst stimulation at ∼120° of the SLM θ resulted in little potentiation (Fig. 4*C*), while burst stimulation at ∼340° resulted in apparent LTD (Fig. 4*D*). In the group, RAD burst stimulation was delivered at approximately three times the threshold intensity (86.3 ± 9.3 μA, *n* = 25).

**Figure 5.**
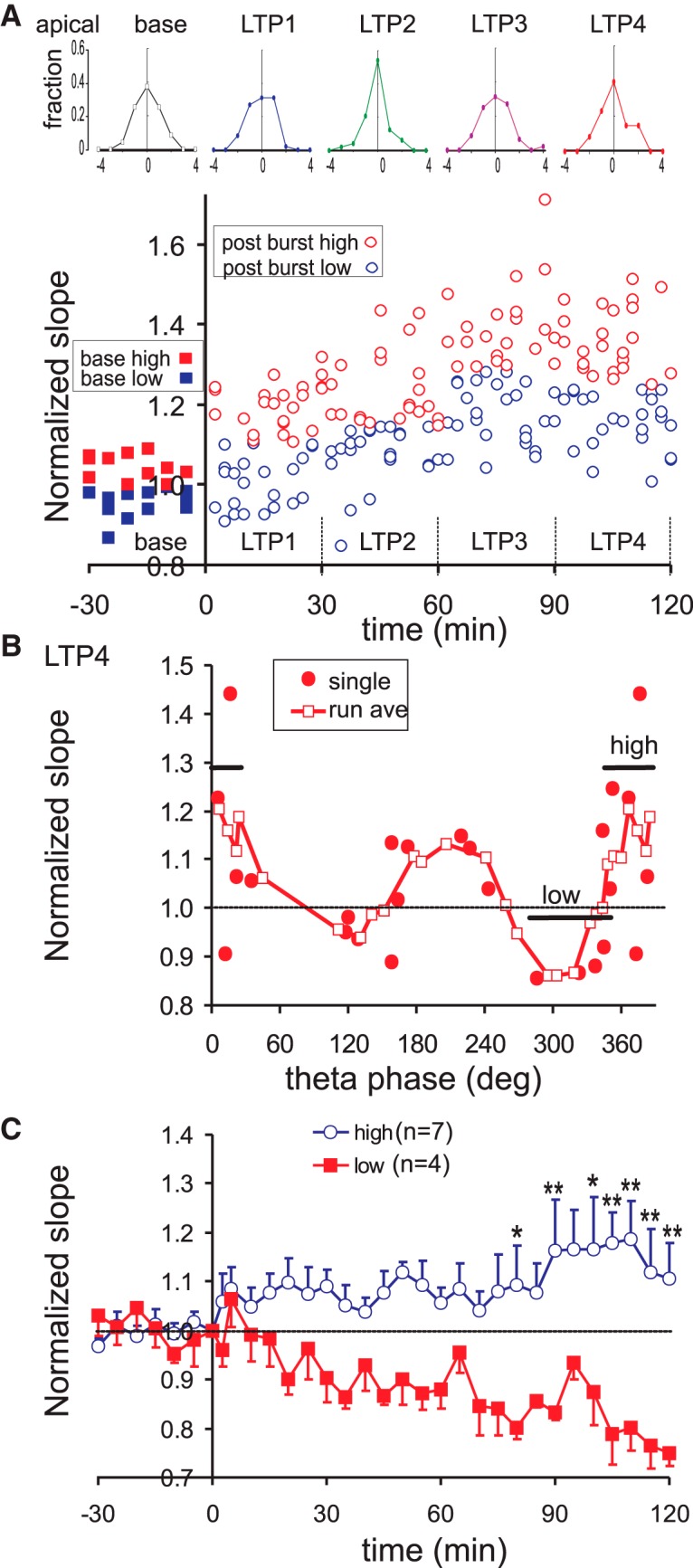
Variability of apical dendritic response in time, and relation of apical dendritic late LTP with SLM θ phase. ***A***, Time course of apical dendritic LTP illustrated by single-sweep response in a representative rat (same as rat shown in Fig. 4*B*); single sweep responses were sorted as above (high) or below (low) the median during a 30-min time periods (LTP1–LTP4). Top row shows the frequency distribution at baseline and LTP1–LTP4; vertical axis labeled as fraction of occurrence in different bins away from the mean (horizontal axis), with 1 bin = SD during baseline. Frequency distribution did not change significantly with time periods. ***B***, Normalized slope of the apical dendritic ES of single rats (filled circles), and running phase-average of the normalized slope of three to four rats (run ave; open squares), are plotted with the θ phase at burst onset; data points at θ phase 360–390° are the same as those at 0–30°. ***C***, Mean ± SEM of the normalized ES slope was plotted for the high and low group indicated in ***B***. High and low groups were significantly different, as shown by two-way repeated measured ANOVA [*F*_(1,9)_ =13.8, *p* < 0.01], with *post hoc* differences indicated by *p < 0.05 and **p < 0.01.

Frequency distribution of single-sweep evoked responses following RAD stimulation, evaluated in 30-min blocks, did not show significant changes with time in the rat shown (Fig. 5*A*). In a group of rats, the SD of the apical dendritic sink slopes during the baseline was 0.10 ± 0.01 (*n* = 25), which was not significantly different from SD of the basal dendritic sink slopes of 0.086 ± 0.005 (*n* = 26). The SD did not change among the five groups of 30-min blocks, from baseline to LTP1–LTP4, as indicated by a lack of group effect [*F*_(4,96)_ = 0.74, *p* > 0.7; one-way repeated measures ANOVA]. The stability of the SD suggests that brain state variability did not critically affect the mean apical dendritic responses in 30-min blocks.

The plot of apical dendritic LTP4 with θ phase at burst onset indicates two maxima, each preceded by a minimum (Fig. 5*B*). The right pair of minimum and maximum was labeled as low and high conditions in Figure 5*B*. The average time course of the normalized response reveals a rising LTP in the high group, which is different from the low group that shows a decreasing response with time (Fig. 5*C*).

### Relation between LTP and θ phase

**Figure 6.**
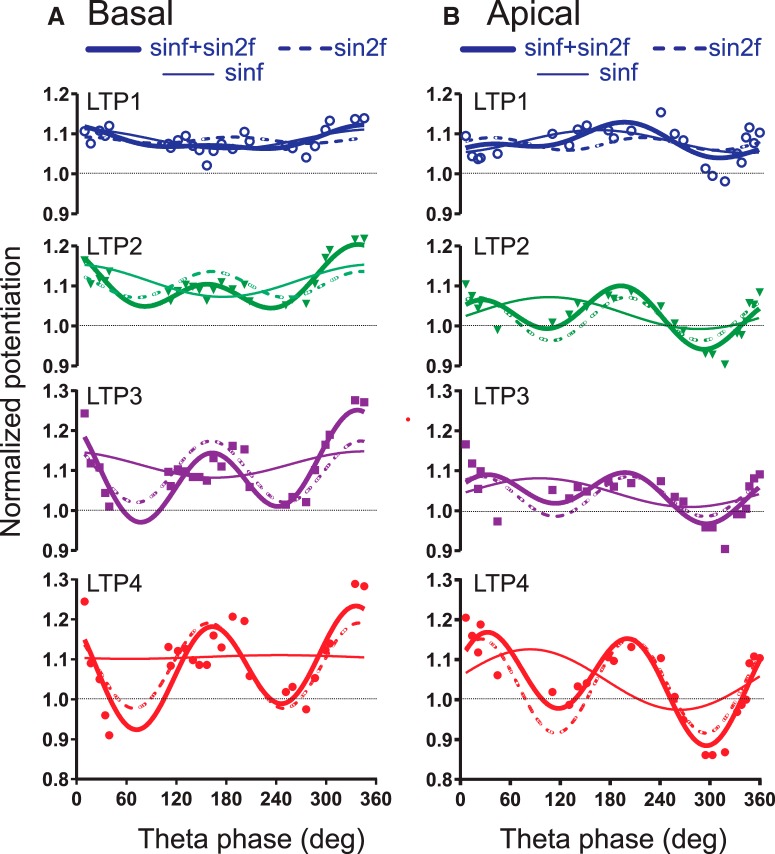
Basal and apical dendritic potentiation in relation to SLM θ phase of burst onset, averaged for different time periods post-bust. ***A***, Individual normalized basal dendritic slopes (symbols) were averaged (three- or four-point running average) with phase, and then curve-fitted with a single 1st harmonic plus constant (sinf), a 2nd harmonic plus constant (sin2f), or the sum of 1st and 2nd harmonics plus constant (sinf+sin2f). From top to bottom, LTP1 (averaged for 0–30 min post-burst), LTP2 (30–60 min), LTP3 (60–90 min), and LTP4 (90–120 min post-burst). Other than LTP1, data points of all other periods show a good curve fit by a 2nd harmonic alone, or the sum of 1st and 2nd harmonics. ***B***, Same as ***A*** for the apical dendritic response. Again, LTP2–LTP4 show good fit by a 2nd θ harmonic, and better by a sum of 1st and 2nd θ harmonics. The 2nd θ harmonic modulation of either basal or apical LTP increased with time post-burst.

The run-averaged basal dendritic LTP with θ phase (at burst onset) was curve-fitted by the sum of θ 1st and 2nd harmonics. The basal dendritic ES showed short-term potentiation (LTP1 at 0–30 min post-burst) of ∼8.3%, irrespective of the θ phase of burst stimulation onset. Basal dendritic LTP1 was modulated by a θ 1st harmonic, and not significantly modulated by a 2nd harmonic (Fig. 6*A*; Table 1). Late basal dendritic LTP shows increasing modulation by the 2nd as compared to the 1st harmonic (Fig. 6*A*; Table 1). The relation of basal dendritic LTP4 with θ phase was moderately well fitted by a sum of 1st and 2nd harmonics (*R* ^2^ = 0.574; Table 1; Fig. 6*A*), or by a 2nd harmonic alone (*R* ^2^ = 0.485), but not by a 1st harmonic alone (*R* ^2^ = 0.001; Fig. 6*A*; Table 1). The fitted 2nd harmonic peaked at 160° and 340° θ phase (Fig. 6*A*).

**Table 1.** Parameters (mean ± SEM) of curve fit of phase-averaged normalized ES slope (y) versus θ phase (x) for (A) basal and (B) apical dendritic excitation

Parameter	LTP1	LTP2	LTP3	LTP4
A				
A0	1.083 ± 0.005	1.102 ± 0.005	1.096 ± 0.009	1.082 ± 0.014
A1	0.026 ± 0.0065	0.050 ± 0.007	0.057 ± 0.012	0.042 ± 0.021
A2	97.13 ± 17.26°	109.7 ± 9.06°	130.4 ± 13.57°	162.1 ± 27.0°
B1	0.012 ± 0.009	0.051 ± 0.010	0.102 ± 0.015	0.125 ± 0.024
B2	58.71 ± 15.11°	67.12 ± 3.54°	64.99 ± 3.11°	65.53 ± 4.02°
B1/A1	0.47	1.02	1.78	3.0
*R* ^2^ (df = 20)	0.465*	0.791**	0.73**	0.574**
B				
A0	1.065 ± 0.008	1.012 ± 0.018	1.028 ± 0.006	1.045 ± 0.011
A1	0.032 ± 0.013	0.0178 ± 0.0083	0.006 ± 0.014	0.0474 ± 0.0154
A2	–135.9 ± 17.83°	–94.16 ± 35.11°	–112.4 ± 142.3°	–16.6 ± 31.25°
B1	0.038 ± 0.010	0.070 ± 0.009	0.066 ± 0.013	0.130 ± 0.012
B2	18.57 ± 8.83°	24.27 ± 4.03°	19.81 ± 6.47°	18.46 ± 3.0°
B1/A1	1.18	3.93	10.96	5.79
*R* ^2^ (df = 19)	0.472*	0.773**	0.579**	0.877**

The data were fitted by a sum of 1st and 2nd θ harmonics with equation y = [A0 + A1 sin (π* (x + A2)/180) + B1 sin (π* (x + B2)/90)], where A0 = phase-independent constant, A1 = 1st harmonic modulation, A2 = 1st harmonic phase shift, B1 = 2nd harmonic modulation, and B2 = 2nd harmonic phase shift. *R* ^2^ describes the goodness of the fit with degrees of freedom (df). LTP was averaged for different periods after bursting: LTP1 (0–30 min), LTP2 (30–60 min), LTP3 (60–90 min), and LTP4 (90–120 min). B1/A1 ratio increased with time;

** *p* < 0.005, ^*^p < 0.05, *R* ^2^ significantly different from random pairing of x and y values.

The run-averaged early potentiation (LTP1) of the apical dendritic sink was not well fitted by θ harmonics. However, the phase relation of LTP2, LTP3, and LTP4 was well fitted by a sum of θ harmonics plus constant (Table 1; Fig. 6*B*), or by a 2nd harmonic alone plus constant (Table 1). In particular, the late apical dendritic LTP4 was well fitted by a sum of harmonics (*R* ^2^ = 0.88; Table 1; Fig. 6*B*), or a 2nd harmonic alone (*R* ^2^ = 0.86), but not by a 1st harmonic alone (*R* ^2^ = 0.12). The fitted 2nd harmonic peaked at 210° and 390° (or 30°) θ phase (Fig. 6*B*).

Apical dendritic LTP versus θ phase function was shifted to the right with respect to the basal dendritic LTP versus θ phase function (Fig. 6). Based on the error estimates from the curve fits, the 2nd harmonic phase delay (B2) was significantly different between apical and basal dendritic LTP, with basal dendritic LTP leading the apical dendritic LTP by 47°. In addition, the constant curvefit parameter A0 was higher for basal dendritic than apical dendritic LTP in all time periods. This indicates that there is a phase-independent enhancement of the average basal dendritic response as compared to the average apical dendritic response (Fig. 6).

The main features of the basal and apical dendritic LTP and their phase relation were the same when alternate measures of LTP (single-sweep sink slopes, sink amplitude or sum of sinks at the rising phase), or of θ phase (see FFT method in Materials and Methods), were used (data not shown).

### Bursting induced LTP during non-θ state

**Figure 7.**
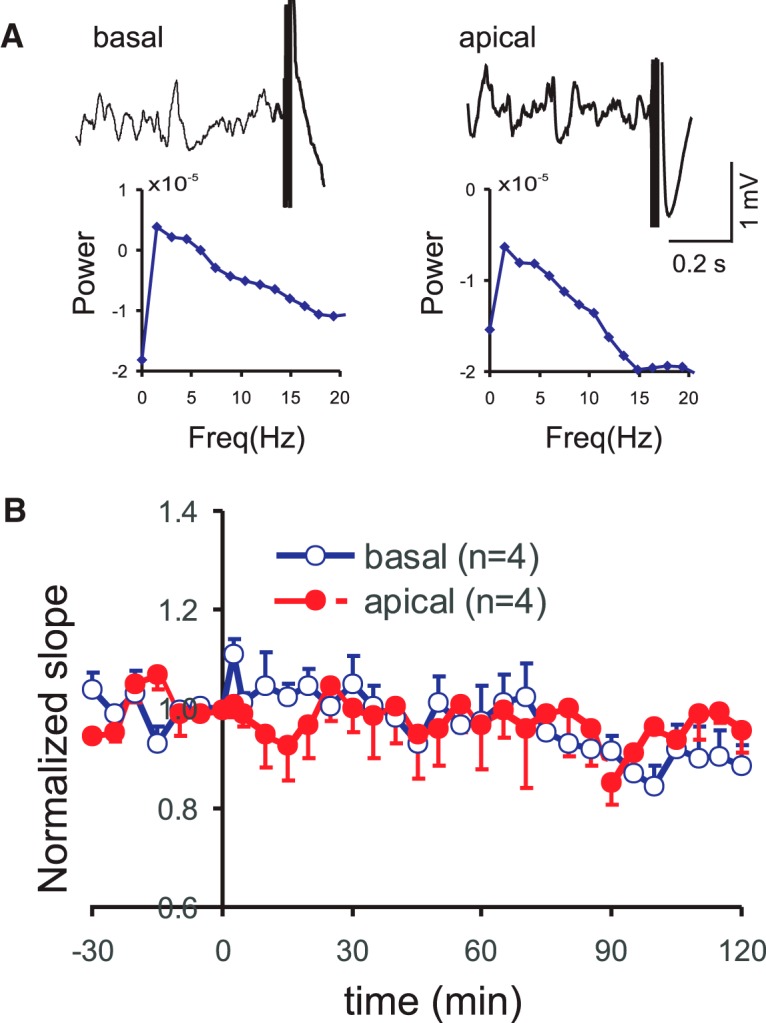
LTP was not induced by burst stimulation during non-θ state. ***A***, left, Example of a burst stimulation of OR during non-θ LFP, in an attempt to induce basal dendritic LTP. Power spectrum shows high amplitude at <2 Hz. Right, Same except for an example of burst stimulation of RAD in attempt to induce apical dendritic LTP. ***B***, Group average of the basal and apical dendritic normalized sink slopes induced by a burst stimulation administered during a non-θ LFP.

In some animals, a burst stimulation was delivered when the spontaneous LFP showed slow waves with no clear θ rhythm (Fig. 7*A*). When burst stimulation was delivered during non-θ LFP, there was a lack of basal dendritic LTP following OR stimulation (*n* = 4), or of apical dendritic LTP following RAD stimulation (*n* = 6; Fig. 7*B*).

### NMDA receptor currents contributed to bursting-induced ESs and LTP

**Figure 8.**
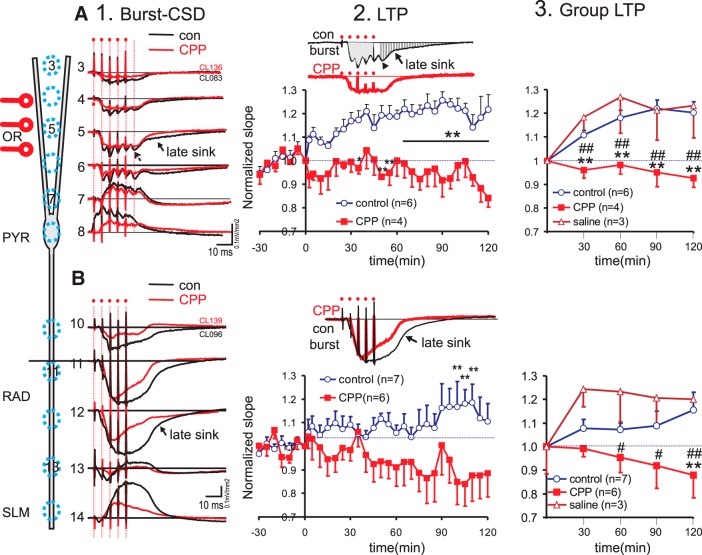
Effect of NMDA receptor antagonist CPP on dendritic bursts and burst-induced LTP induced during the rising phase of distal dendritic θ. ***A***, Single burst stimulation (five pulses at 20-ms intervals, artifacts indicated by dots) of OR evoked a CSD profile of sustained basal dendritic sink at channel 3–6, accompanied by sources near pyramidal cell layer (PYR, channel 8). Profile for a rat after CPP diffusion is overlaid on that from a control (con) rat with no micropipette. At the maximal sink, the duration of the sink was prolonged in con compared to CPP rat (arrow in channel 5), and small spike-like sinks was observed (arrowhead). Column 2 inset shows early integrated sink as gray, and late integrated sink filled with vertical stripe. The time course of the mean ± SEM of the normalized sink slope was shown for the no-pipette control and CPP groups, indicating significant *post hoc* differences at specific time points. Column 3 shows mean ± SEM of burst-induced basal dendritic LTP in three groups of rats (with local micropipette containing CPP, with micropipette containing saline, or control without micropipette); LTP was averaged in blocks of 30 min (e.g., 90- to 120-min period labeled as 120 min). After a significant two-way ANOVA, *post hoc* comparisons between CPP and control (no pipette) groups: #*p* < 0.05, ##*p* < 0.01; CPP versus saline groups: *p < 0.05, **p < 0.01. ***B***, Same as ***A*** except for the apical dendritic LTP following burst stimulation of RAD. Column 1, burst evoked CSDs show sustained apical dendritic sinks in RAD, accompanied by source in SLM. No PSs are obvious during the bursts in control or CPP rat, while the late sink amplitude and sink duration (arrow) were reduced in CPP as compared to control rat. Columns 2 and 3, Apical dendritic LTP was suppressed by CPP as compared to controls.

The CSDs during burst stimulation and the subsequent LTP were assessed after diffusion of NMDA receptor antagonist CPP (1 mM) or saline from a local micropipette, and in a no-drug condition without a micropipette (called the “no-pipette” group). After 90 min of diffusion, a single OR burst stimulation was given at the rising phase of the SLM θ [CPP group 357 ± 15°, *n* = 4; saline group 333.7 ± 17.9°, *n* = 3; no-pipette group 349.8 ± 13.3°, *n* = 6]. The CSDs induced by burst stimulation of OR were different in CPP group as compared no-CPP groups. The early integrated sink within 0–20 ms of burst onset (Fig. 8*A1*) was smaller (*p* < 0.05, Wilcoxon) in the CPP group (*n* = 4) than a combined control group (*n* = 9), which included three rats with saline pipette and 6 rats without pipette. The duration of the sink (until zero crossing) at the maximal basal dendritic sink, and the ratio of the late integrated sink (25 ms from burst onset to zero crossing; Fig. 8*A2*) to the early integrated sink was also significantly smaller in the CPP than the control group (*p* < 0.05, Wilcoxon), suggesting that the late sink was mediated by NMDA receptor-mediated currents. Burst stimulation evoked small PSs (Fig. 8*A1*, arrowhead) with maximal sinks in OR and not PYR (Fig. 8*A1*).

OR burst stimulation did not induce basal dendritic potentiation in the CPP group, illustrated by comparing with the LTP time course in the no-pipette group without CPP (Fig. 8*A2*). LTP averaged across 30-min blocks was used for statistical comparison among the three groups, CPP, saline, and no-pipette groups. Two-way (three groups × four time blocks) repeated measures ANOVA of the basal dendritic LTP showed a significant group effect [*F*_(2,10)_ = 6.66, *p* < 0.01], without time or group × time interaction effects. *Post hoc* Newman–Keuls tests showed a significant difference between the CPP group and the saline or the no-pipette group (Fig. 8*A3*), but no difference between the saline and the no-pipette groups (Fig. 8*A3*).

A RAD-stimulated burst evoked a large apical denritic sink (Fig. 8*B1*), typically larger than the basal dendritic sink evoked by a OR-stimulated burst (Fig. 8*A1*). In the CPP group (*n* = 7) as compared to a combined control group (*n* = 9, with three saline-pipette rats and six no-pipette rats), the RAD burst evoked apical dendritic sink response was significantly reduced in duration (*p* < 0.01, Wilcoxon) and in the ratio of the late integrated sink to the early integrated sink (*p* < 0.05, Wilcoxon), but not in the early integrated sink. Bursting was delivered at the rising phase of the SLM θ in all rats [CPP group 370.4 ± 6.1°, *n* = 7; saline group 361.8 ± 23.7°, *n* = 3; no pipette group 365.7 ± 15.9°, *n* = 6].

In the CPP group, RAD burst stimulation at the rising SLM θ did not induce a significant apical dendritic LTP; the normalized apical dendritic sink actually showed a gradual decline with time (Fig. 8*B2*,*B3*). The decrease in apical dendritic sink was different from the LTP in the saline or the no-pipette group (Fig. 8*B2*,*B3*). Two-way (three groups × four time blocks) repeated measures ANOVA of the normalized apical dendritic sink confirmed a significant group effect [*F*_(2,13)_ = 6.58, *p* < 0.02], without time or group × time interaction effects. *Post hoc* Newman–Keuls tests showed significant difference between the CPP group and the saline or the no-pipette group (Fig. 8*B3*).

### Relation of burst response to LTP

The ratio of the late integrated sink to the early integrated sink did not correlate with the magnitude of LTP at the basal or apical dendrites, when the LTP induced at all θ phases were included. This suggests that the ratio of NMDA to non-NMDA receptor currents does not predict LTP magnitude across all θ phases. At the basal dendrites, the total ES at OR evoked by the five-pulse stimulus burst was negatively correlated with basal dendritic LTP2, LTP3, and LTP4 (correlation coefficients of –0.47, –0.50, and –0.55, respectively, all *p* < 0.02). However, at the apical dendrites, the total ES at RAD evoked by the five-pulse burst was not significantly correlated with apical dendritic LTP1–LTP4.

### Basal dendritic PS varies with θ phase and θ/non-θ states

**Figure 9.**
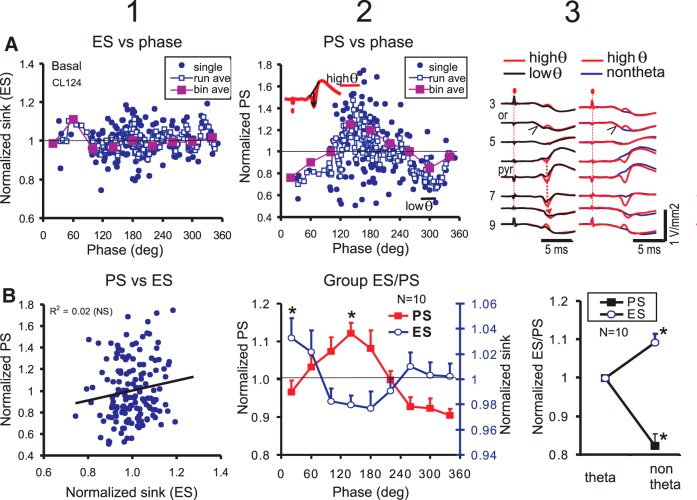
Single-pulse OR stimulation evoked basal dendritic ES and PS as a function of SLM θ phase. ***A***, Normalized ES (column 1) and PS (column 2) amplitude plotted versus SLM θ phase in a representative rat (CL124). ES and PS were plotted for single sweeps following a high-intensity stimulus pulse delivered to OR, and after running average (run ave) and phase-bin average (bin ave). Column 3, Average CSD laminar profiles overlaid with high excitability (high θ) and low excitability (low θ) θ phases (left), and with high θ and non-θ states (right). ***B***, column 1, Scatter plot of normalized PS and ES shows non-significant (NS) linear correlation. Column 2, Mean ± SEM group (*n* = 10) phase-bin averaged PS amplitude shows a maximal excitability at 140° of the SLM θ rhythm, and ES amplitude shows a maximum at 20°; *p < 0.05 from one and all groups with values below 1. Column 3, Average ES slope was significantly (*p < 0.05) higher but PS amplitude significantly lower during a non-θ state as compared to θ state.

To evaluate the change in excitatory synaptic transmission in relation to θ phase, single stimulus pulses were delivered at different phases of the SLM θ or during a non-θ LFP. Single-pulse stimulation of OR evoked ES slope at the basal dendrites (ES; Fig. 9*A1*) and a PS at PYR (Fig. 9*A2*). The responses of one rat, normalized by the respective mean measure, show considerable variation at a particular θ phase. Thus, each measure was grouped into phase bins of 40° interval, and this reveals high ES at the 60° bin and maximal PS amplitude at the 140° bin (Fig. 9*A2*). Phase-selected averages of the evoked CSD response confirmed a 2-fold larger amplitude for PS evoked during “high θ” compared to “low θ” condition, with no difference in the ES slopes between high θ and low θ conditions (Fig. 9*A3*). The PS sink evoked by OR stimulation apparently started at proximal OR/pyramidal cell layer, and then propagated to the apical dendrites (Fig. 9*A3*, left column, downward arrow). A scatter plot of the normalized PS amplitude with normalized ES slope for all single sweeps shows a non-significant linear correlation in this rat (Fig. 9*B1*), which was found in most rats in the group (*n* = 10 rats).

In the group of 10 rats, the θ phase-bin average of the basal dendritic PS amplitude (Fig. 9*B2*) was significantly different among bins [*F*_(8,72)_ = 6.14, *p* < 0.0001, one-way repeated measures ANOVA]. The bin-averaged PS peaked at 140° θ phase, and troughed at 340° (Fig. 9*B2*). As an alternate phase estimate, a running average of PS versus phase was made, and then subjected to non-linear curve fitting. A sum of 1st and 2nd sinusoidal harmonics gave a good curve fit of the PS-phase data (*R* ^2^ = 0.55 ± 0.06, *n* = 10 rats), and showed a dominance of the 1st over the 2nd harmonic (Table 2). The peak PS occurred at 131.3 ± 11.1° (*n* = 10) of the 1st harmonic sine wave, consistent with the bin average peak at 140°. Curve-fit with only a 1st harmonic plus constant also gave a good fit (*R* ^2^ = 0.49 ± 0.06) with an estimated peak at 128.7 ± 10.7° (*n* = 10).

**Table 2.** Mean ± SEM of parameters for basal and apical dendritic single-pulse response (*n* = 10 rats, each group)

	Basal	Apical	*p*
Stimulus Intensity (μA)	402 ± 49	251 ± 66	NS
ES onset (ms)	3.63 ± 0.21	3.20 ± 0.19	NS
ES slope (mV/mm^2^)	–163 ± 21	–383 ± 33	<0.001
ES slope ratio (non-θ/θ)	1.09 ± 0.02	1.20 ± 0.04	<0.05
PS peak latency (ms)	5.60 ± 0.16	5.65 ± 0.26	NS
PS peak amplitude (mV/mm^2^)	299 ± 56	549 ± 118	<0.03
PS amplitude ratio (non-θ/θ)	0.83 ± 0.03	1.64 ± 0.12	<0.001
Curve-fit of PS-θ phase with sum of harmonics			
A0	0.972 ± 0.013	0.989 ± 0.004	NS
A1	0.132 ± 0.026	0.098 ± 0.0151	NS
A2	–41.3 ± 11.1°	–49.90 ± 8.23°	NS
B1	0.042 ± 0.007	0.046 ± 0.008	NS
B2	22.8 ± 19.1°	6.7 ± 21.0°	NS
B1/A1	0.36 ± 0.05	0.60 ± 0.15	NS
R^2^	0.55 ± 0.06	0.53 ± 0.07	NS

PS sink amplitude and ES slope (in CSD units of mV/mm^2^) were evoked at a stimulus intensity of 1.5 times the PS threshold. The average amplitude/slope measures were determined during a θ state (averaged across all phases) or non-θ state. PS amplitude and ES slope, and their respective ratio in non-θ-to-θ state were statistically different between basal and apical dendritic measures. Scatter plot of single sweep PS (y) with SLM θ phase (x) at stimulus onset, were fitted by equation y = [A0 + A1 sin (π* (x + A2)/180) + B1 sin (π* (x + B2)/90)], where A0 = constant, A1 = 1st harmonic modulation, A2 = 1st harmonic phase shift, B1 = 2nd harmonic modulation, and B2 = 2nd harmonic phase shift. *R* ^2^ describes the goodness of fit. Probability (*p*) of equality between basal and apical groups was tested by unpaired Wilcoxon; NS, non-significant, *p* > 0.05.

Using bin averages, the basal dendritic ES slope was shown to be significantly different among θ phase bins [*F*_(8,72)_ = 2.33, *p* < 0.03, one-way repeated measures ANOVA], with a peak at the 20° phase bin and a minimum at ∼180° (Fig. 9*B2*). Single sinusoidal curve-fit of the ES slope to θ relation gave a relatively poor fit (*R* ^2^ = 0.28 ± 0.08), and high phase variability of the estimated peak at 61.2 ± 24.8° (*n* = 10).

Single-pulse responses were recorded during periods of non-θ LFPs, which were intermingled with θ LFPs. Average ES slope and PS amplitude were different between non-θ and θ states, as shown by a representative rat (Fig. 9*A3*, right column) and the group average (Fig. 9*B3*). The average basal-dendritic ES slope was ∼10% higher in a non-θ state (Fig. 9*B3*), while the average PS amplitude (evoked by OR stimulation) decreased ∼20% during non-θ as compared to θ state (Fig. 9*B3*). The ratio of θ power to low-δ (integrated 1–2 Hz) power was confirmed to be significantly different between θ and non-θ states (data not shown).

### Apical dendritic PS varies with θ phase and θ/non-θ states

**Figure 10.**
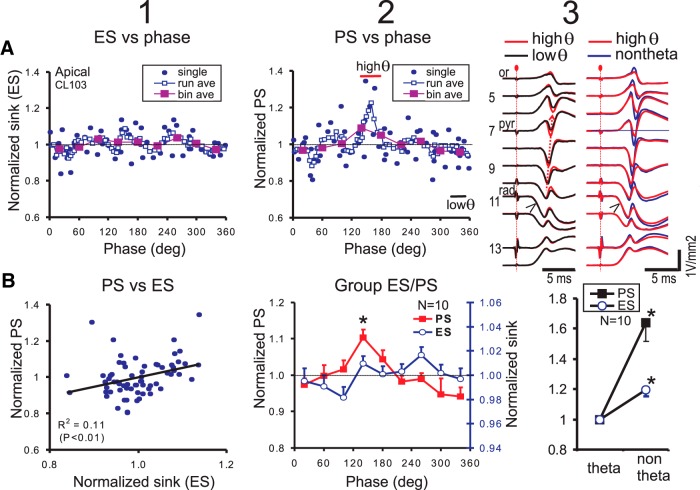
Single-pulse RAD stimulation evoked apical dendritic ES and PS as a function of θ phase. ***A***, Normalized ES (column 1) and PS amplitude (column 2) plotted versus SLM θ phase in a representative rat (CL103). ES and PS were plotted for single sweeps (evoked by a high-intensity stimulus pulse delivered to RAD), and after running average (run ave) and phase-bin average (bin ave). Column 3, Average CSD laminar profiles overlaid with high excitability (high θ) and low excitability (low θ) θ phases (left), and with high θ and non-θ states (right). ***B***, column 1, Scatter plot of normalized PS and ES shows a small but significant linear correlation. Column 2, Mean ± SEM group (*n* = 10) phase-bin averaged PS amplitude shows maximal excitability at 140° of the SLM θ rhythm, while ES amplitude shows no significant relation to θ phase; *p < 0.05, different from one and all groups with mean <1. Column 3, Average ES slope and PS amplitude during non-θ state was significantly (*p < 0.05) higher than that during θ state.

Following single-pulse stimulation of RAD, the single-pulse evoked normalized ES slope at the apical dendrites (ES; Fig. 10*A1*) and normalized PS amplitude at PYR (Fig. 9*A2*) of one rat show variation at a particular θ phase. Both running and bin averages of the PS in the representative rat gave a PS maximum at 130–150° θ phase (Fig. 10*A2*). The phase-selected average CSD traces show larger PS during the high θ (∼140° phase) as compared to the low θ (∼340° phase) condition (Fig. 10*A3*, right column). The PS evoked by RAD stimulation apparently started in proximal RAD and propagated to PYR (Fig. 10*A3*, upward arrow). The θ phase-bin average of the apical dendritic PS amplitude (Fig. 10*B2*) was significantly different among bins [*F*_(8,72)_ = 3.71, *p* < 0.002, one-way repeated measures ANOVA], with a maximum at 140°, and a minimum at 300–340° (Fig. 10*B2*). Curve fitting of the running PS average with the sum of 1st and 2nd harmonic sine waves gave an estimated apical dendritic PS peak at 139.9 ± 8.3° (*n* = 10; Table 2), consistent with the bin averaged peak of 140°.

The normalized ES slope (Fig. 10*A1*) showed low variation across phase in running and bin averages of a representative animal. There was no clear difference in the apical dendritic ES slope (Fig. 10*A3*, arrow head) evoked during high θ versus low θ (labeled in Fig. 10*A2*) conditions. The bin-averaged apical dendritic ES slope did not show a significant variation with θ phase [*F*_(8,72)_ = 1.05, *p* > 0.4, one-way repeated measures ANOVA; Fig. 10*B2*]. Analysis of the pEPSP slope also indicated no statistically significant modulation by θ phase (data not shown). A scatter plot of the normalized PS amplitude versus normalized ES slope showed a small but significant correlation for the rat illustrated (Fig. 10*B1*), and for six of 10 rats in the group; the remaining four rats showing no significant ES-PS correlation.

Both OR and RAD single-pulse stimulation evoked a PS maximum at ∼140° and a PS minimum at ∼340° θ phase. PS amplitude modulation by θ phase was not different between OR and RAD stimulus groups, shown by a non-singificant (*p* > 0.4) group effect in a two-way (group × phase bins) ANOVA, or by the curvefit parameters derived from individual rats (Table 2). Higher responses (ES slope or PS peak) were evoked following single-pulse RAD stimulation than OR stimulation (Table 2).

Apical dendritic ES slope and PS amplitude were different during a non-θ state as compared to a θ state. The average apical-dendritic ES slope was ∼20% higher in a non-θ than a θ state (Fig. 10*B3*), while the average PS amplitude (evoked by RAD stimulation) was ∼60% higher during a non-θ state as compared to a θ state (Fig. 10*B3*). The ES slope increased from a θ state to a non-θ state, and this increase was larger for apical than basal dendritic ES (Table 2).

### θ Phase modulation of γ and ripples activity

**Figure 11.**
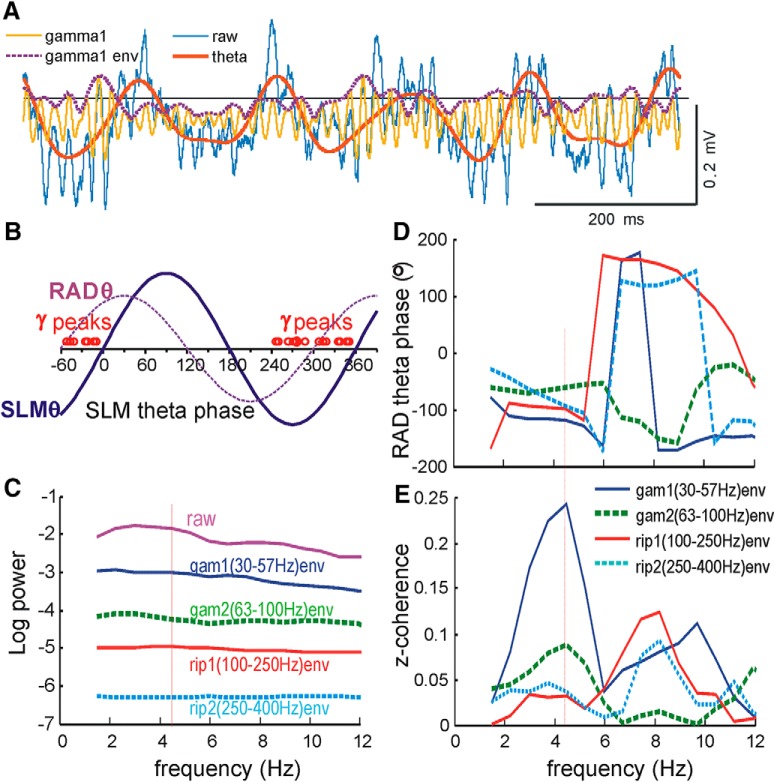
Cross-frequency coupling between θ and low γ (30–57 Hz) and other frequency bands of the LFPs. ***A***, Illustration of a single segment of LFP recorded at CA1 RAD, shown as raw (unfiltered) signal, θ (0–8 Hz filtered), γ1 or gam1 (30–57 Hz filtered), with gam1 envelope (env) peaking at a phase earlier than the θ peaks. ***B***, Distribution of the estimated phase of the 30- to 57-Hz γ (gam1) peaks (γ) in 17 rats with reference to SLM θ phase, illustrated with a schematic RAD θ. ***C***, Logarithmic power spectrum, average of nine segments of 8192 points, showing mean power versus frequency (0–12 Hz) for the raw unfiltered signal, and the four frequency bands (gam1, gam2, rip1, and rip2). Cross-frequency phase spectrum (with respect to RAD θ frequency; ***D***) and coherence z-transform (z-coherence) spectrum (***E***) of the same segments, showing peak coherence at 4.47 Hz (vertical line) with corresponding phase around –100° of the RAD θ phase for all four frequency bands.

γ And ripples LFPs were known to be modulated by θ phase. to show the phase modulation of LTP in relation of that of LFPs, phase-amplitude coupling of the γ and ripples activity to θ activity was evaluated in rats that showed apical dendritic LTP (Fig. 11). An electrode in RAD showing maximal apical ES response was selected for cross-frequency coupling analysis using coherence and phase spectra (Methods). The envelope of four LFP bands, low (30–57 Hz) and high (63–100 Hz) γ, low (100–250 Hz) and high (250–400 Hz) ripples, showed decreasing power at a fixed θ frequency (Fig. 11*C*; Table 3). The envelope of low-γ activity was observed to peak on the rising phase (∼0°) of the RAD θ (Fig. 11*A*), which corresponded to ∼300° of the SLM θ (Fig. 11*B*). Cross spectral analysis of the low-γ envelope and θ activity revealed a cross-frequency coherence peak at 0.24 and phase of –118° (with the RAD θ) at 4.47 Hz (Fig. 11*D*,*E*). The cross-frequency phase remained at near –100° of the RAD θ (Fig. 11*D*), at the cross-frequency coherence peaks for high γ, low and high ripples (Fig. 11*E*). The group mean cross-frequency θ-low γ coherence peak was 0.14 ± 0.2 (*n* = 17 rats), with RAD θ phase –80.1 ± 12.7° (*n* = 17). Mean cross-frequency peak coherence decreased from low γ to high γ, low ripples, and high ripples activity (Table 3). Because of low coherence, frequency bands other than low γ showed a scattered phase distribution with relatively high SEs (Table 3).

**Table 3.** Coherence and phase measures of the cross-frequency coupling of different frequency bands to θ phase frequency analyzed at a RAD electrode

	Low γ (30–57 Hz)	High γ (63–100 Hz)	Low ripples (100–250 Hz)	High ripples (250–400 Hz)
Peak log power	–1.13 ± 0.06	–2.18 ± 0.04	–2.97 ± 0.05	–4.00 ± 0.10
Phase (RAD θ)	–80.1 ± 12.7°	–53.5 ± 23.8°	–58.2 ± 20.7°	–73.0 ± 21.6°
z-coherence	0.14 ± 0.02	0.069 ± 0.016	0.086 ± 0.019	0.057 ± 0.012
Number	17	17	19	16

For each frequency band, a z-transform coherence (z-coherence) peak of >0.005 at a θ (2.9–6 Hz) frequency was required for inclusion. The mean peak theta frequency of the unfiltered signal (*n* = 19 rats) was 3.61 ± 0.10 Hz, with power of 0.46 ± 0.06 log units; zero log power is 1 mV^2^/Hz. Phase was analyzed using the RAD θ as reference.

## Discussion

The present study provides strong evidence that a hippocampal θ rhythm modulates spike excitability and synaptic plasticity in CA1 area at different phases of a θ cycle. By using CSD analysis to separate basal and apical dendritic ESs, and a reliable θ phase reference at the CA1 distal dendrites (SLM), we found that a single burst stimulation of OR induced basal dendritic LTP optimally at two phases, rising (∼340°) and falling phases (∼160°) of the SLM θ. Burst stimulation of RAD also optimally induced apical dendritic LTP at the rising (∼30°) and falling phases (∼210°) of the SLM θ. Late (30–120 min) LTP of both basal and apical dendritic synapses were modulated by a θ 2nd harmonic. PSs evoked by single-pulse OR or RAD stimulation showed maximal excitability at ∼140° of the SLM θ, OR-evoked basal dendritic sink peaked at ∼20°, while γ and ripples power peaked at ∼300° of the SLM θ, different from the LTP modulation with phase.

### θ Phase-dependent LTP

**Figure 12.**
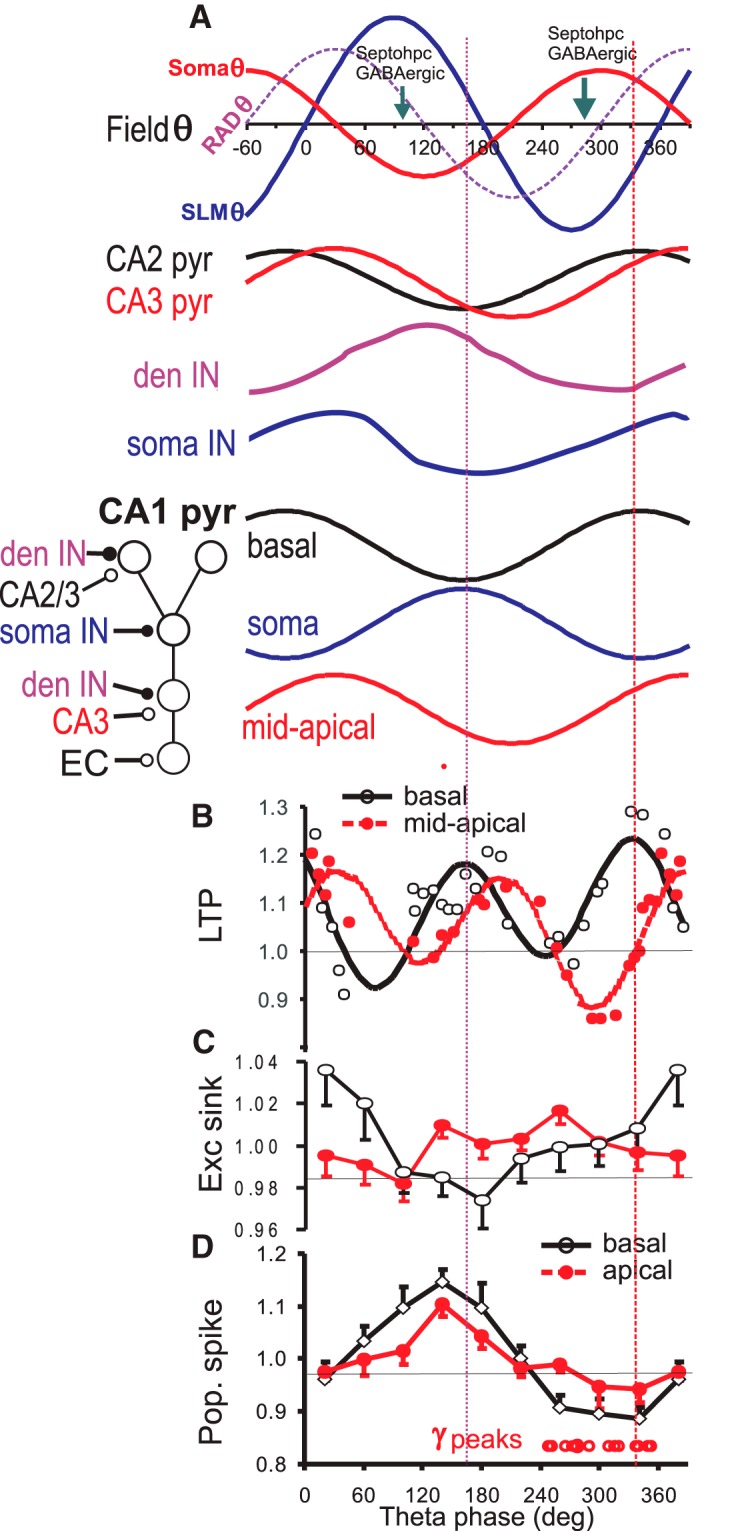
Summary of θ phase dependent LTP and excitability. ***A***, Schematic diagram of θ field potentials recorded at the pyramidal cell body layer (Soma θ), RAD (RAD θ), and SLM (SLM θ); soma θ and RAD θ are assumed to be 150° and 60° phase advanced, respectively, to SLM θ. Times of maximal firing of septohippocampal (Septohpc) GABAergic neurons (Borhegyi et al., 2004) are indicated by downward arrows. θ Phase dependent firing rate of pyramidal cells (pyr) in CA2 and CA3, with a range of firing phase (after Mizuseki et al., 2009), dendrite-targeting (den IN) and soma-targeting interneurons (soma IN; after Klausberger et al., 2003, 2004) are shown, CA1 pyramidal cells are shown with basal dendritic, soma and mid-apical (RAD) dendritic compartments, with inferred membrane potential traces in relation to SLM θ. Input from entorhinal cortex (EC) is not shown. Normalized LTP (***B***), normalized excitatory (exc) sink slope (***C***), and normalized evoked population (pop.) spike (***D***) following excitation of basal and mid-apical dendritic (RAD) synapses. γ Power (γ; 30–57 Hz) peaks are indicated by the row of open circles near the horizontal axis showing θ phase, with a range from 0° to 390°; 360–390° is the same as 0–30°.

The study substantially extends previous studies that maximal mid-apical dendritic LTP (evoked by RAD stimulation) was induced when burst stimulations occurred at the positive phase of the RAD θ (Hölscher et al., 1997; Hyman et al., 2003). In our study, maximal mid-apical dendritic LTP at the rising phase of the SLM θ fell well within the positive phase of the RAD θ, which was ∼60° phase advanced to the SLM θ (Figs. 1, 12*A*). The use of SLM instead of RAD for the θ phase reference in the present study allows higher phase precision since θ phase can vary up to 120° within RAD (Fig. 1*B2*). In addition, we reported another peak of the apical dendritic LTP at ∼210° of the SLM θ (Fig. 12*A*), and two peaks within a single θ cycle suggest modulation by a θ 2nd harmonic (Fig. 6). The use of single burst instead of multiple bursts avoided the issue of whether multiple bursts, used in previous studies, fell on the same θ phase.

We presented original results on the modulation of basal dendritic LTP by θ phase. Burst stimulation of OR induced LTP optimally at two phases, rising (∼340°) and falling (∼160°) phases of the SLM θ. The biphasic θ modulation was similar to that of the RAD-evoked apical dendritic LTP, confirming modulation by a θ 2nd harmonic. However, the basal dendritic LTP-θ phase function was 47° phase advanced to the apical dendritic LTP-phase function (Fig. 12*B*), i.e., shifted by ∼1/8th of the fundamental θ cycle.

Brain state (non-θ vs θ LFP) was not determined for the responses recorded in the LTP experiments, but it was suggested not to affect the 30-min block LTP results. The present results showed that the basal and apical dendritic ES slopes were ∼10% and ∼20% larger in non-θ state as compared to θ state. Frequency distribution of single-pulse evoked ES slopes indicated that its variability (SD) did not change significantly across 30-min time blocks. Since single-sweep ES sink slope amplitude was correlated with brain (θ/non-θ) state, a frequency distribution of slope amplitudes would approximate the distribution of θ and non-θ states, thus suggesting that the latter distribution did not change significantly across 30-min time blocks. A relatively constant ratio of θ to non-θ state in 30-min time blocks is consistent with the cycling of θ and non-θ states in urethane-anesthetized rats with a period of ∼11 min (Clement et al., 2008). Our own data showed that under the same conditions used for LTP recording, non-θ state occurred 40.5 ± 2.8% in 15- to 30-min blocks (*n* = 15 rats). The low variability (SEM/mean ratio) in the occurrence of non-θ (or θ) state confirms that 15- to 30-min averaging was adequate to remove brain state dependency.

LTP induced during the rising phase of the SLM θ was found to be sensitive to NMDA receptor blockade. NMDA receptor antagonist CPP blocked both basal and apical dendritic LTP induced at the rising phase of SLM θ (Fig. 8). We suggest that burst stimulation induces glutamate release, which opens up NMDA receptors when it coincides with θ-rhythmic postsynaptic depolarization at the dendrites. NMDA receptor-sensitive LTP was typically found after low-intensity tetanic stimulation in CA1 (Capocchi et al., 1992; Roth and Leung, 1995; Leung and Shen, 1999; Habib and Dringenberg, 2009).

A late ES during burst stimulation was preferentially suppressed by CPP, suggesting that the late sink was mainly mediated by NMDA receptors. Thus, the ratio of late-integrated sink to early-integrated sink is interpreted to approximate the ratio of NMDA to non-NMDA receptor mediated currents. Across different θ phases, the ratio of the late to early integrated sink did not correlate significantly with the magnitude of LTP, suggesting that the sink ratio alone does not predict θ phase-dependent LTP. Furthermore, the total (early + late) ES evoked by the burst, which represents the total excitatory currents, did not predict LTP. At the basal dendrites, the burst-evoked total ES was negatively correlated with basal dendritic LTP2, LTP3, and LTP4. However, at the apical dendrites, the burst-evoked total ES was not significantly correlated with apical dendritic LTP at any time period. ES s may be reduced by a high level of dendritic membrane depolarization that reduces excitatory electromotive force but facilitates basal dendritic LTP.

Maximal LTP likely results from maximal dendritic depolarization evoked by the burst stimulation, which adds onto the dendritic membrane potential before the burst. Network activity determines the level of dendritic membrane potential, and for CA1 pyramidal cells, afferent activity comes from local inhibitory interneurons and distant (CA2, CA3, and entorhinal layer 3) excitatory neurons. Disinhibition of pyramidal cells is expected to follow low firing of dendrite-targeting interneurons, including O-LM interneurons and bistratified cells, at ∼300° of the SLM θ rhythm (Fig. 12*A*; Klausberger et al., 2003, 2004). In addition, maximal firing of CA3 and CA2 pyramidal cells, at –30–90° SLM θ phase (Mizuseki et al., 2009; Oliva et al., 2016), excites the CA1 pyramidal cells at the basal and apical dendrites. The period of maximal LTP at the CA1 basal and apical dendrites falls within the phase range of firing of population of CA2 and CA3 pyramidal cells (Fig. 12*A*). Previous models of generation of a CA1 θ rhythm incorporate rhythmic CA3 proximal excitation at the apical dendrites (Brankack et al., 1993; Kocsis et al., 1999; Buzsáki, 2002), and rhythmic distal excitatory driving by the entorhinal cortex (Leung, 1984; Mizuseki et al., 2009); however, a LTP dependence on θ 2nd harmonic was not predicted by these models.

The prominent modulation of LTP in CA1 by a θ 2nd harmonic is an original finding. A 2nd θ harmonic has been observed in spontaneous LFPs and depolarization-induced membrane potential oscillations (Leung et al., 1982; Leung and Yu, 1998; Sheremet et al., 2016), but seldom alone without a dominant 1st harmonic. Hippocampal driving at two separate phases in a θ cycle may originate from septohippocampal GABAergic neurons, with short- and long-burst neurons firing respectively at the trough and peak of the PYR θ (Borhegyi et al., 2004). Septohippocampal GABAergic neurons strongly drive the hippocampal θ rhythm (Boyce et al., 2016), assisted by septohippocampal cholinergic (Vandecasteele et al., 2014) and glutamatergic neurons (Robinson et al., 2016). Also, single and burst firing in CA1 neurons occurred at two separate θ phases (Mizuseki et al., 2012), consistent with inferred maximal somatic and dendritic depolarization.

θ Phase-dependent LTP was equally robust for basal and apical dendritic synapses. A single burst stimulation at a similar stimulus intensity did not induce LTP during non-θ LFPs. θ Rhythm may provide effective disinhibition at optimal times, which is necessary for θ phase-dependent LTP, and disinhibition may not be present during a non-θ state. Tetanic stimulation of longer duration (not targeted to a specific θ phase) induced LTP more readily at the basal than apical dendritic synapses (Kaibara and Leung, 1993; Arai et al., 1994; Roth and Leung, 1995; Li et al., 2016).

### Dependence of evoked PS and ES on θ phase

Evoked PS amplitude manifested a single maximum at ∼140° of the SLM θ rhythm, following either basal or apical dendritic excitation. This is consistent with maximal PS excitability in CA1 at the falling phase of a dentate gyrus θ rhythm in walking rats, following stimulation of the ventral hippocampal commissure (Rudell et al., 1980). However, maximal apical dendritic pEPSP during the falling phase of the apical dendritic θ rhythm (Wyble et al., 2000; Schall et al., 2008) was not found in the present study (Fig. 10*B2*). This was likely because of our use of high stimulus intensities to evoke a PS, and assessment of ES/pEPSP at short latencies (<4 ms). We reported an original result that the basal dendritic ES was significantly modulated by θ phase, showing a small (2–3%) increase at ∼20°, and a small decrease at ∼200° phase of the SLM θ (Fig. 9*B2*).

Linear correlation between ES and PS, after basal or dendritic excitation, was found to be low. This suggests that the controlling factor for PS was not ES, but perhaps other factors such as dendritic depolarization or somatic/axonal inhibition. The amplitude of PS at PYR layer does not distinguish the origin of the PS, which was suggested to originate from the basal dendrites after OR stimulation, and from the apical dendrites after RAD stimulation (Figs. 9*A3*, 10*A3*; Kloosterman et al., 2001). Decrease in firing of soma-directed interneurons, i.e., basket cells to the soma and axoaxonic cells to the initial segments (Klausberger et al., 2003, 2004), is followed by maximal disinhibition (depolarization) of the soma and initial segment, resulting in a period of maximal PS amplitude (Fig. 12*A*,*D*). Low spontaneous pyramidal cell firing is accompanied by a period of low PS excitability at 300–360° phase (Fig. 12*A*,*D*).

A basal dendritic LTP peak appeared at 120–190° θ phase, near the ∼140° phase of the maximal spike excitability (Figs. 3*B*, 12*B*); the apical dendritic mid-cycle LTP peak occurred somewhat later at 180–210° (Figs. 5*B*, 12*B*). We suggest that the basal dendritic LTP may be facilitated by a spread of somatic depolarization to the proximal dendrites. Spike backpropagation (Magee and Johnston, 1997) and spike depolarizing afterpotentials (Fung et al., 2016) have been suggested to facilitate dendritic LTP. However, dendritic hyperpolarization is large at a θ phase of 120–210° (Fig. 12*A*), and we speculate that dendritic I_h_ or Ca^2+^ T-currents (Magee and Johnston, 1997; Hu et al., 2009) may assist the transition from hyperpolarization to depolarization.

Intracellular recordings of hippocampal CA1 pyramidal cells in urethane-anesthetized rats showed that somatic and dendritic membrane potentials were out of phase (Ylinen et al., 1995; Kamondi et al., 1998), perhaps the same as the out-of-phase occurrence of maximal spike excitability and maximal LTP at the rising phase of the SLM θ. We suggest that basal dendritic membrane may show a phase-advanced depolarization as compared to the apical dendritic membrane, in accordance with a ∼50° phase lead of the basal dendritic LTP peak with respect to the apical dendritic LTP peak. The reason for a phase advance of basal versus apical dendritic depolarization is not known, but excitatory inputs to the basal versus apical dendrites, such as those from CA2 versus CA3 pyramidal cells (Fig. 12*A*), may provide the phase shifts.

### State-dependent excitability and LTP

ES and PS following dendritic stimulation were different between θ versus non-θ states, as reported previously. During a θ as compared to a non-θ state, apical dendritic PS was smaller while basal dendritic PS was larger, while apical and basal dendritic ES was both smaller but still different from each other (Table 2). These results were consistent with previous reports of the state-dependent basal (Leung and Péloquin, 2010) and apical dendritic pEPSP or ES (Leung, 1980; Herreras et al., 1988; Wyble et al., 2000; Schall et al., 2008).

Results so far indicated that burst stimulation did not induce LTP during a non-θ state. However, LTP induction at fixed times in relation to components of the non-θ LFP, such as slow oscillation (Schall et al., 2008), respiration-coupled slow rhythm (Lockmann et al., 2016), or sharp waves (Buzsáki et al., 1983), had not been studied.

### θ Phase modulation of γ activity and LTP

We found that the amplitude of low γ (30–57 Hz) LFP recorded at RAD peaked at ∼300° of the SLM θ, which coincided with the positive peak of the soma θ rhythm. The θ-γ phase relation was consistent with that reported in behaving rats (Belluscio et al., 2012). The phase of maximal low γ power at ∼300° was different from the phase of maximal apical or basal dendritic LTP, but near the trough of apical dendritic LTP (Fig. 12). In the present study, the mean θ phase for maximal power of high γ, low and high ripples power was similar to that of low γ power, but with higher phase variability.

### Functional significance of θ phase-dependent LTP

Basal dendritic afferents are derived from CA3a, CA3b, and CA2 cells, while apical dendritic afferents are derived from CA3b and CA3c cells (Ishizuka et al., 1990; Li et al., 1993; Shinohara et al., 2012; Kohara et al., 2014). The types of information carried by basal and apical afferents to CA1 are likely different. CA2 may represent emotional, social and temporal aspects, while CA3 represents spatial aspects of memory (Hitti and Siegelbaum, 2014; Mankin et al., 2015). By having different phases for maximal LTP at basal and apical dendritic trees, separate windows are maintained for integration and encoding of different types of information. Basal and apical dendritic LTP also have different molecular mechanisms (Leung and Péloquin, 2010; Li et al., 2016; Brzdak et al., 2018).

Separate memory encoding and retrieval phases are proposed within a θ cycle, with separate encoding (maximal LTP) and retrieval (maximal CA1 response) phases (Hasselmo et al., 2002; Kunec et al., 2005). The present results suggest that a second encoding period (LTP) can occur in CA1, near the time of maximal soma excitability. Douchamps et al. (2013) showed that a novel environment induced up to 50° θ phase advance of neuronal firing peak from the PYR θ trough, which we interpret to fall within our mid-cycle LTP peak at 160–210° SLM θ phase, rather than the LTP peak at the θ rising phase. Spike timing-dependent plasticity has been suggested to occur by shifting spike firing ∼20 ms near the phase of inhibitory conductance minimum (Kwag and Paulsen, 2009), which corresponds to the period of maximal excitability near the mid-cycle LTP peak.

## Conclusions

The present study only used urethane-anesthetized rats, and a quantitative hippocampal LTP to θ phase relation remains to be shown in behaving rats. θ Frequency is lower in urethane-anesthetized rats than behaving rats. However, PS excitability in CA1 appears to be similar in urethane-anesthetized and behaving rats (above), and θ 2nd harmonic and gradual θ phase shift in CA1 were observed under urethane anesthesia in the present study.

To our knowledge, it is the first time that synaptic plasticity was studied across all phases of a complete brain oscillation cycle. The present study highlights the multiple ways a brain oscillation can modulate synaptic transmission, γ activity, spike excitability, and synaptic plasticity. The θ rhythm modulates the processing of neural signals in the entorhinal cortex-hippocampus network, and controls the encoding and retrieval of memory by discrete dendritic compartments.

10.1523/ENEURO.0236-18.2018.supplement Supplementary Table 1-1 Parameters (mean ± SE) of curve fit by a single sine wave of the phase-averaged normalized LTP (y) versus theta phase (x) for A. basal and B. apical dendritic excitation. LTP fitted with single sinusoid, either first harmonic y = [A0 + A1 sin (π* (x + A2) /180], or second harmonic y = [B0 + B1 sin (π* (x + B2) /90]. Download Table 1-1, DOC file

## Synthesis

Reviewing Editor: Menno Witter, Norwegian University of Science and Technology Decisions are customarily a result of the Reviewing Editor and the peer reviewers coming together and discussing their recommendations until a consensus is reached. When revisions are invited, a fact-based synthesis statement explaining their decision and outlining what is needed to prepare a revision will be listed below. The following reviewer(s) agreed to reveal their identity: Clayton Dickson, Steven Fox. Note: If this manuscript was transferred from JNeurosci and a decision was made to accept the manuscript without peer review, a brief statement to this effect will instead be what is listed below.

This is an important study on the modulation of the induction of long-term potentiation (LTP) and synaptic/suprathreshold excitability in hippocampal area CA1. Both reviewers concur that you presented a well planned and carried out series of experiments and that your data are really important and should be published. However, the potential impact of your study is greatly reduced due to a less than optimal organization of the text and figures. Before we can accept your paper, it needs serious rewriting, and the reviewers made a number of constructive suggestions with respect to the figures as well. Simplification and clarification of the manuscript in general are important. The present form might keep it from being appreciated for its importance by newcomers to the field. That would be a tragedy, because we see your paper as a first serious step toward understanding the mechanisms that underlie these major hippocampal phenomena.

Reviewer 1

This study examined the modulation of the induction of long-term potentiation (LTP) and synaptic/suprathreshold excitability in hippocampal area CA1 in an in vivo urethane anesthetized rat preparation as related to the phase of ongoing spontaneous theta oscillation. It is a highly systematic study with a number of critical elements that set it well apart (and well above) previous work on this topic. I will list them here:

1) Current source density (CSD) analysis. Measurements of extracellular field LTP and excitatory synaptic activity involved the actual computation of extracellular current movement based on simultaneous multichannel local field potential (LFP) profiles recorded from linear probes penetrating through the basal to apical dendritic axis of CA1 pyramidal neurons. Previous studies have typically relied on single monopolar LFP recordings which could vary in their location from the actual synaptic sites studied in the dendritic layer in which they occur.

2) Full sampling of the complete phase of ongoing theta as measured at a consistent and reliable location. Previous studies have typically only studied the peak positive and/or negative phases of the ongoing theta rhythm, often recorded at different (and often suboptimal) dendritic locations within and across experiments.

3) The use of a single quick burst (5 pulses at 200 Hz) at a defined phase of theta to elicit LTP, whereas previous studies have used multiple bursts across different theta cycles which could introduce variation in the results due to theta phase jitter.

Modulation of synaptic and input-output functions across the theta phase is highly important aspect of understanding hippocampal physiology. It is well known that hippocampal excitability varies across the theta cycle and that there is an exquisitely tuned symphony of cellular discharges that is paced by this population rhythm. Given its proposed importance for encoding memory and facilitation synaptic potentiation, it is critical to understand how the dynamics of theta rhythm may alter the functional properties of the hippocampal circuit and its relevance to the neurobiology of memory.

Although this is a highly exhaustive, impressive and important study, it is severely limited by its complexity in presentation. There are many examples in the text, figures, and captions in which explicit simplifications would greatly help to parse out the many different (and often difficult) effects that make up the many aspects of this study. I would like to make it clear at this point that I am very supportive of this work but have some (what I hope to be taken as) constructive criticisms for the authors to improve the presentation of these important data.

Results are summarized as below:

1) Single burst stimulation of fibres in stratum oriens (denoted as ‘basal’) induces LTP optimally when timed to the initial rising (∼340°) and falling phases (∼160°) of the theta rhythm recorded at the level of stratum lacunosum moleculare (SLM). This biphasic modulation appears to follow both the theta fundamental and 1st harmonic frequencies.

2) Single burst stimulation of fibres in stratum radiatum (denoted as ‘apical’) induces LTP optimally when timed to the later halves of both the rising (∼30°) and falling (∼210°) phases of the theta rhythm recorded at the level of stratum lacunosum moleculare. As with ‘basal’ LTP, this biphasic modulation appears to follow both the theta fundamental and 1st harmonic frequencies.

3) The same single burst stimulation of fibres in either location (‘basal’ or ‘apical’) does not result in LTP when delivered during a non-theta state.

4) When single burst stimulation was delivered at its preferential theta phase in both ‘basal’ and ‘apical’ conditions, it was blocked by the NMDA receptor antagonist CPP.

5) Evoked population spike amplitudes elicited by single pulse stimulation of either ‘basal’ or ‘apical’ fibres were modulated maximally at ∼140° of the ongoing theta cycle phase.

6) Maximal evoked sinks for ‘basal’ stimulation using single pulses was at ∼20°.

7) Maximal ongoing high frequency modulation (gamma to ripple frequencies) occurred at ∼300° of SLM theta.

8) Conclusion: Theta modulation of dendritic synaptic plasticity and both dendritic and and somal excitability temporally compartmentalizes maximal responding in these different zones, likely due to the functional phasing of both excitatory and inhibitory inputs to these regions.

Related to the presentation, it would be extremely helpful to have a similar systematic organization of these conclusions in the discussion section to allow the reader to follow the logic of the authors.

General Comments:

Presentation and writing style: The manuscript reads a bit choppily and could use a fair degree of copy-editing. The writing style somewhat detracts from the otherwise excellent experimentation. Of particular importance for the authors to consider would be the front end of the document, especially the abstract and significance statement. A summary figure regarding prior research would be helpful to organize information in the introduction. Figure arrangements could benefit from a more systematic, simplified, and explicit layout that makes the take-home point. Reliance on figure captions to understand the figures should be at an absolute minimum. This is certainly not the case in the present version. Another piece of advice would be to explicitly state the purpose of each different analysis within the results section (for example, explicitly stating the rationale for doing the high-frequency- gamma/ripple- to theta phase evaluation that is presented in Figure 10.

Relevance of theta phase and its appropriate measurement: The context of the phase and power profile of theta rhythm through the CA1 axis and an appreciation for why the SLM is the best site for determining theta phase would really benefit from an explicit figure showing this outright - with example raw LFP traces together with the phase/power profile shown in Figure 1E. Otherwise, this important point risks being lost to the non-theta initiate. In addition, this would make it plain that the phase indications being used in the text and figures are with respect to a sine wave (i.e. starting at 0 amplitude at 0°.

State and evoked potential amplitude: While the authors agree that brain state (in addition to theta phase) has a significant modulatory effect on the evoked potential responses, this modulation is not explicitly acknowledged in the figures by only showing EP amplitudes as simply a function of time. Indeed, the baseline conditions explicitly acknowledge that both theta and non-theta states have been collapsed in the averages presented. A more definitive solution would be to explicitly separate states both in the baseline and post stimulation cases. One explicit solution would be to overlay an indication of state changes across time in the explicit example shown in Figure 1B_2 where there is a clear 15 minute cycle which is undoubtedly related to spontaneous state changes under urethane. This would help to alleviate concerns of variability in the synaptic measures.

Statistical evaluations: The phase-related modulation of LTP and evoked potential responses seem to require a more systematic statistical evaluation, especially given the variance shown in single and multiple experiments. One solution is to determine the confidence limits for baseline and average measures across time (preferably organized by the state in which they were tested) and to explicitly show the data with respect to these limits. Otherwise, it will be suspect as to whether data are truly different from each other as a repeated measure.

Simplification and explicit enunciation: This manuscript contains a great deal of complicated data and analysis. It would be highly beneficial to systematically elaborate on some of them graphically. For example, a visual representation of the SLM theta waveform (or representative sine wave) with a graphical phase marking on each figure panel that shows date with respect to this demarcation would make it absolutely plain that the phase delineation is consistent with a sine wave starting at 0 degrees. Otherwise, this is left to the reader to decipher. As a more concrete example of a lack of explicitness, the order of panels in Figure 1 seems to be backwards -It would seem that the theta data would be the first thing to demonstrate (see above) and then next, the CSD of the LTP experiment.

Organization: Certain elements do not flow well or even logically. As an explicit example the raw data for ‘apical’ LTP (Figure 4) should appear before the averaged data appears in Figure 3. As well, the discussion should be organized around the major findings (in order of presentation). This is not the case presently. Given that LTP is the first aspect of the results, then the discussion should follow this order.

Related to the writing style, simplification and organization, it appears that the major problem with this manuscript is that it needs to be read and re-read again in order to parse out and then fully understand the main findings. If these are made more explicit and simplified in the first place in a logical order then this would be immensely better. As a suggestion of explicitness, and related to having more obvious figure layouts and content, the use of abbreviations and ‘short-hand’ terminology (‘basal’ and ‘apical’ being examples which could be written out long-hand as ‘;st oriens’ and ‘st radiatum’ stimulation conditions}.

Specific Comments:

Model of theta modulation: Although the last figure is a welcome summary of the hypothesized modulation results, this could be made more explicit using an illustration of the separate pyramidal compartments with models of dendritic and somal inputs together with their appropriate phases. This would allow a more systematic evaluation of the idea being presented.

Reviewer 2

This paper reports that the NMDAR-dependent LTP in CA1 induced by a stimulus burst mimicking the complex spikes of hippocampal pyramidal cells shows two maxima at phases of the hippocampal theta cycle separated by about 180 degrees, whereas the population spikes evoked by single pulses peak at a single phase. The two phase-maxima for producing proximal apical dendritic LTP are delayed about 50 degrees from those for basal dendritic LTP, but the population spike maxima for those two sites occur at the same phase. The authors conclude that somatic disinhibition is the likely mechanism accounting for the ‘excitability’ peak for both sets of afferents, whereas the LTP maxima reflect more localized dendritic depolarizations, with a variety of possible origins. Sorting out the various possibilities will require additional experiments suggested by the results of this very important first step.

This is a very comprehensive series of experiments that were carefully planned and executed. The precision of the phase angles reported is much improved over earlier studies by the use of an entire depth profile of theta phase in each experiment, and LTP induction using a single 20ms stimulus burst. This precision is especially valuable for allowing comparison with other published (or future) data to aid in explanation of the candidate mechanisms underlying these novel observations.

There are, however, some issues with the presentation.

To begin with the abstract, clarification is essential. It should help to guide the general reader through this complex paper. For example ‘apical’ is vague, because it could mean SLM. ‘Radiatum afferents’ is clearer. Definition of 0 degrees should appear here. Bringing the term ‘second harmonic’ in at this point just makes it seem too technical. Reporting the two peak phases for basal LTP is followed by describing a ‘phase delay’ between them and the radiatum LTP, then calling the ‘minor’ peak a ‘second harmonic’. Instead, it would be much simpler to say that there were two phases of theta for strong basal and radiatum LTP; ∼180 and ∼330 degrees after the SLM positive-going zero-crossing for basal LTP and ∼20 and ∼230 degrees for radiatum LTP. It seems to me that the two peaks on the cycle are of comparable magnitude.

It might be helpful if the order in Results and Discussion were similar. Perhaps even reordering the Results to present ‘excitability’ first, then LTP.

This reviewer has some concern about the author's theta criterion of Q&gt;1 (p.12, line 209) for including animals. In figures and averages the Qs are much higher. In imagining what a Q = 1.1 spectrum might look like, I worry whether I would call the associated time domain signal ‘theta’.

On p. 20, line 384 the authors use the phrase ‘LTP decline’ to refer to a decreasing response with time. In order to have an LTP decline you would first have to have LTP. Earlier in the manuscript they use ‘apparent LTD’ to refer to a similar slow decrease in response. That seems a more appropriate descriptor.

In the section on gamma (p.24, lines 474-476) the gamma envelope is reported as having a phase of -118 degrees with respect to the theta recorded (at the same depth?) in radiatum and the reader is referred to Fig 10B, but I think that single number may apply to the data in Fig 10A. Fig 10B shows a distribution of gamma peak results whose mean is presumably the value reported at the end of line 477. That group mean phase is reported as -80.1 degrees. It should be made abundantly clear in the text that these are NOT numbers on the phase scale used everywhere else. Better yet, perhaps it should be reported as “cross phase spectra showed that the gamma peak occurs at ∼272 degrees with respect to SLM theta” (at line 476), and group mean... ∼300 degrees (at line 477). This is similar to what was already done on p. 26, line 530. The phase labels of Fig 10B are nonstandard for this manuscript and therefore terribly confusing. I believe that it would be greatly improved by showing both SLM theta and RAD theta exactly like it is shown in Fig 11A, with tick labels that represent the theta phase numbers used everywhere else in the document. (By the way, adding to the confusion is mislabeling of the next to last tick mark in Fig 10B as 390, it should be 420 on this non-standard scale.)

On p. 31, lines 627-628, the authors state: “To our knowledge, it is the first time that synaptic plasticity was studied as a function of the phase of a brain oscillation in mammals in vivo.” This confuses me greatly, since the authors cite several other in vivo studies of LTP vs. theta phase. They should make it clear what they mean by this assertion or delete the sentence.

Picky stuff:

Fig 3A has a key element with the label ‘group’. Elsewhere ‘run ave’ is used. The latter is clear and helpful, the former is vague and mysterious.

Fig 8A3: again, the key is not helpful. The left half is about high and low response phases and the right half is about high quality theta and non-theta. It needs better key labels.

Fig 10D and E do not seem to be mentioned in the text and even the legend is very brief. It is not clear why they are here.

## References

[B1] Arai A, Black J, Lynch G (1994) Origins of the variations in long-term potentiation between synapses in the basal versus apical dendrites of hippocampal-neurons. Hippocampus 4:1–9. 10.1002/hipo.450040103 8061748

[B2] Belluscio MA, Mizuseki K, Schmidt R, Kempter R, Buzsáki G (2012) Cross-frequency phase-phase coupling between q and g oscillations in the hippocampus. J. Neurosci 32:423–435. 10.1523/JNEUROSCI.4122-11.2012 22238079 PMC3293373

[B3] Bland BH, Colom LV (1993) Extrinsic and intrinsic properties underlying oscillation and synchrony in limbic cortex. Prog Neurobiol 41:157–208. 8332751 10.1016/0301-0082(93)90007-f

[B4] Bliss TVP, Collingridge G, Morris R (2007) Synaptic plasticity in the hippocampus In: The hippocampus book (Andersen P, Morris R, Amaral D, Bliss T, O'Keefe J, eds), pp 343–434. Oxford: Oxford University Press.

[B5] Borhegyi Z, Varga V, Szilágyi N, Fabo D, Freund TF (2004) Phase segregation of medial septal GABAergic neurons during hippocampal theta activity. J Neurosci 24:8470–8479. 10.1523/JNEUROSCI.1413-04.2004 15456820 PMC6729892

[B6] Boyce R, Glasgow SD, Williams S, Adamantidis A (2016) Causal evidence for the role of REM sleep theta rhythm in contextual memory consolidation. Science 352:812–816. 10.1126/science.aad5252 27174984

[B7] Brankack J, Stewart M, Fox S (1993) Current source density analysis of the hippocampal theta rhythm: associated sustained potentials and candidate synaptic generators. Brain Res 615:310–327. 8364740 10.1016/0006-8993(93)90043-m

[B8] Brzdak P, Wójcicka O, Zareba-Koziol M, Minge D, Henneberger C, Wlodarczyk J, Jerzy W, Mozrzymas W, Wójtowicz T (2018) Synaptic potentiation at basal and apical dendrites of hippocampal pyramidal neurons involves activation of a distinct set of extracellular and intracellular molecular cues. Cereb Cortex. Advance online publication. Retrieved December 8, 2017. doi: 10.1093/cercor/bhx324 10.1093/cercor/bhx324 29228131

[B9] Buzsáki G (2002) Theta oscillations in the hippocampus. Neuron 33:325–340. 11832222 10.1016/s0896-6273(02)00586-x

[B10] Buzsáki G, Leung LS, Vanderwolf CH (1983) Cellular bases of hippocampal EEG in the behaving rat. Brain Res Rev 6:139–171. 10.1016/0165-0173(83)90037-1 6357356

[B11] Capocchi G, Zampolini M, Larson J (1992) Theta burst stimulation is optimal for induction of LTP at both apical and basal dendritic synapses on hippocampal CA1 neurons. Brain Res 591:332–336. 1359925 10.1016/0006-8993(92)91715-q

[B12] Clement EA, Richard A, Thwaites M, Ailon J, Peters S, Dickson CT (2008) Cyclic and sleep-like spontaneous alternations of brain state under urethane anaesthesia. PLoS One 3:e2004. 10.1371/journal.pone.0002004 18414674 PMC2289875

[B13] Douchamps V, Jeewajee A, Blundell P, Burgess N, Lever C (2013) Evidence for encoding versus retrieval scheduling in the hippocampus by theta phase and acetylcholine. J Neurosci 33:8689–8704. 10.1523/JNEUROSCI.4483-12.2013 23678113 PMC3715394

[B14] Dragoi G, Buzsáki G (2006) Temporal encoding of place sequences by hippocampal cell assemblies. Neuron 50:145–157. 10.1016/j.neuron.2006.02.023 16600862

[B15] Freund TF, Buzsáki G (1996) Interneurons of the hippocampus. Hippocampus 6:347–470. 10.1002/(SICI)1098-1063(1996)6:4&amp;lt;347::AID-HIPO1&amp;gt;3.0.CO;2-I 8915675

[B16] Fung TK, Law C, Leung LS (2016) Associative spike-timing dependent potentiation of the basal dendritic excitatory synapses in the hippocampus in vivo. J Neurophysiol 115:3264–3274. 10.1152/jn.00188.2016 27052581 PMC4942267

[B17] Habib D, Dringenberg HC (2009) Alternating low frequency stimulation of medial septal and commissural fibers induces NMDA-dependent, long-lasting potentiation of hippocampal synapses in urethane-anesthetized rats. Hippocampus 19:299–307. 10.1002/hipo.20507 18853436

[B18] Hasselmo M, Bodelón C, Wyble B (2002) A proposed function for hippocampal theta rhythm: separate phases of encoding and retrieval of prior learning. Neural Comput 14:793–817. 10.1162/089976602317318965 11936962

[B19] Herreras O, Solís JM, Herranz AS, Martín del Río R, Lerma J (1988) Sensory modulation of hippocampal transmission. II. Evidence for a cholinergic locus of inhibition in the Schaffer-CA1 synapse. Brain Res 461:303–313. 3179719 10.1016/0006-8993(88)90260-0

[B20] Hitti FL, Siegelbaum SA (2014) The hippocampal CA2 region is essential for social memory. Nature 508:88–92. 10.1038/nature13028 24572357 PMC4000264

[B21] Hölscher C, Anwyl R, Rowan MJ (1997) Stimulation on the positive phase of hippocampal theta rhythm induces long-term potentiation that can be depotentiated by stimulation on the negative phase in area CA1 in vivo. J Neurosci 17:6470–6477. 9236254 10.1523/JNEUROSCI.17-16-06470.1997 PMC6568346

[B22] Hu H, Vervaeke K, Graham LJ, Storm JF (2009) Complementary theta resonance filtering by two spatially segregated mechanisms in CA1 hippocampal pyramidal neurons. J Neurosci 29:14472–14483. 10.1523/JNEUROSCI.0187-09.2009 19923281 PMC6665813

[B23] Huerta PT, Lisman JE (1995) Bidirectional synaptic plasticity induced by a single burst during cholinergic theta oscillation in CA1 in vitro. Neuron 15:1053–1063. 7576649 10.1016/0896-6273(95)90094-2

[B24] Hyman JM, Wyble BP, Goyal V, Rossi CA, Hasselmo ME (2003) Stimulation in hippocampal region CA1 in behaving rats yields long-term potentiation when delivered to the peak of theta and long-term depression when delivered to the trough. J Neurosci 23:11725–11731. 14684874 10.1523/JNEUROSCI.23-37-11725.2003 PMC6740943

[B25] Ishizuka N, Weber J, Amaral DG (1990) Organization of intrahippocampal projections originating from CA3 pyramidal cells in the rat. J Comp Neur 295:580–623. 10.1002/cne.902950407 2358523

[B26] Kahana MJ, Seelig D, Madsen JR (2001) Theta returns. Curr Opin Neurobiol 11:739–744. 11741027 10.1016/s0959-4388(01)00278-1

[B27] Kaibara T, Leung LS (1993) Basal versus apical dendritic long-term potentiation of commissural afferents to hippocampal CA1: a current-source density study. J Neurosci 13:2391–2404. 8501513 10.1523/JNEUROSCI.13-06-02391.1993 PMC6576486

[B28] Kamondi A, Acsády L, Wang X, Buzsáki G (1998) Theta oscillations in somata and dendrites of hippocampal pyramidal cells in vivo: activity-dependent phase-precession of action potentials. Hippocampus 8:244–261. 10.1002/(SICI)1098-1063(1998)8:3&amp;lt;244::AID-HIPO7&amp;gt;3.0.CO;2-J 9662139

[B29] Klausberger T, Somogyi P (2008) Neuronal diversity and temporal dynamics: the unity of hippocampal circuit operations. Science 321:53–57. 10.1126/science.1149381 18599766 PMC4487503

[B30] Klausberger T, Magill PJ, Márton LF, Cobden PM, Buzsáki G, Somogyi P (2003) Brain-state- and cell-type-specific firing of hippocampal neurons in vivo. Nature 421:844–848. 10.1038/nature01374 12594513

[B31] Klausberger T, Márton LF, Baude A, Roberts JDB, Magill P, Somogyi P (2004) Spike timing of dendrite-targeting bistratified cells during hippocampal network oscillations in vivo. Nat Neurosci 7:41–47. 10.1038/nn1159 14634650

[B32] Kloosterman F, Peloquin P, Leung LS (2001) Apical and basal orthodromic population spikes in hippocampal CA1 *in vivo* show different origins and patterns of propagation. J Neurophysiol 86:2435–2444. 10.1152/jn.2001.86.5.2435 11698533

[B33] Kocsis B, Bragin A, Buzsáki G (1999) Interdependence of multiple theta generators in the hippocampus: a partial coherence analysis. J Neurosci 19:6200–6212. 10.1523/JNEUROSCI.19-14-06200.1999 10407056 PMC6783086

[B34] Kohara K, Pignatelli M, Rivest AJ, Jung HY, Kitamura T, Suh J, Frank D, Kajikawa K, Mise N, Obata Y, Wickersham IR, Tonegawa S (2014) Cell type-specific genetic and optogenetic tools reveal hippocampal CA2 circuits. Nat Neurosci 17:269–279. 10.1038/nn.3614 24336151 PMC4004172

[B35] Kunec S, Hasselmo ME, Kopell N (2005) Encoding and retrieval in the CA3 region of the hippocampus: a model of theta-phase separation. J Neurophysiol 94:70–82. 10.1152/jn.00731.2004 15728768

[B36] Kwag J, Paulsen O (2009) The timing of external input controls the sign of plasticity at local synapses. Nat Neurosci 12:1219–1221. 10.1038/nn.2388 19734896

[B37] Larson J, Lynch G (1986) Induction of synaptic potentiation in hippocampus by patterned stimulation involves two events. Science 232:985–988. 3704635 10.1126/science.3704635

[B38] Leung LS (1980) Behaviour-dependent evoked potentials in the hippocampal CA1 region of the rat. I. Correlation with behavior and EEG. Brain Res 198:95–117. 7407597 10.1016/0006-8993(80)90347-9

[B39] Leung LS (1984) Model of gradual phase shift of theta rhythm in the rat. J Neurophysiol 52:1051–1065. 10.1152/jn.1984.52.6.1051 6097652

[B40] Leung LS (1998) Generation of theta and gamma rhythms in the hippocampus. Neurosci Biobehav Rev 22:275–290. 9579318 10.1016/s0149-7634(97)00014-6

[B41] Leung LS (2010) Field potential generation and current source density analysis In: Electrophysiological recording techniques (Vertes RP, Stackman RW, eds), NeuroMethods Vol15, pp 1–26. Clifton, NJ: Humana Press.

[B42] Leung LS, Yim CY (1986) Intracellular records of theta rhythm in hippocampal CA1 cells of the rat. Brain Res 367:323–327. 3008923 10.1016/0006-8993(86)91611-2

[B43] Leung LS, Yu H (1998) Theta-frequency resonance in hippocampal CA1 neurons in vitro demonstrated by sinusoidal current injection. J Neurophysiol 79:1592–1596. 10.1152/jn.1998.79.3.1592 9497437

[B44] Leung LS, Shen B (1999) N-methyl-D-aspartate receptor antagonists are less effective in blocking long-term potentiation at apical than basal dendrites in hippocampal CA1 of awake rats. Hippocampus 9:617–630. 10.1002/(SICI)1098-1063(1999)9:6&lt;617::AID-HIPO2&gt;3.0.CO;2-6 10641754

[B45] Leung LS, Shen B (2004) Glutamatergic synaptic transmission participates in generating the hippocampal EEG. Hippocampus 4:510–525. 10.1002/hipo.10199 15224986

[B46] Leung LS, Péloquin P (2010) Cholinergic modulation differs between basal and apical dendritic excitation of hippocampal CA1 pyramidal cells. Cereb Cortex 20:1865–1877. 10.1093/cercor/bhp251 19926699

[B47] Leung LWS, Lopes da Silva FH, Wadman WJ (1982) Spectral characteristics of the hippocampal EEG in the freely moving rat. Electroenceph Clin Neurophysiol 54:203–219. 6179745 10.1016/0013-4694(82)90162-6

[B48] Li SB, Du D, Hasan MT, Köhr G (2016) D4 receptor activation differentially modulates hippocampal basal and apical dendritic synapses in freely moving mice. Cereb Cortex 26:647–655. 10.1093/cercor/bhu229 25270308

[B49] Li XG, Somogyi P, Ylinen A, Buzsáki G (1993) The hippocampal CA3 network: an in vivo intracellular labeling study. J Comp Neur 338:1–29. 10.1002/cne.903390204 8300905

[B50] Lockmann ALV, Laplagne DA, Leão RN, Tort ABL (2016) A respiration-coupled rhythm in the rat hippocampus independent of theta and slow oscillations. J Neurosci 36:5338–5352. 10.1523/JNEUROSCI.3452-15.2016 27170130 PMC6601803

[B51] Magee JC, Johnston D (1997) A synaptically controlled, associative signal for Hebbian plasticity in hippocampal neurons. Science 275:209–213. 8985013 10.1126/science.275.5297.209

[B52] Mankin EA, Diehl GW, Sparks FT, Leutgeb S, Leutgeb JK (2015) Hippocampal CA2 activity patterns change over time to a larger extent than between spatial contexts. Neuron 85:190–201. 10.1016/j.neuron.2014.12.001 25569350 PMC4392894

[B53] Martin SJ, Grimwood PD, Morris RGM (2000) Synaptic plasticity and memory: an evaluation of the hypothesis. Ann Rev Neurosci 23:649–711. 10.1146/annurev.neuro.23.1.649 10845078

[B54] Mizuseki K, Sirota A, Pastalkova E, Buzsáki G (2009) Theta oscillations provide temporal windows for local circuit computation in the entorhinal hippocampal loop. Neuron 64:267–280. 10.1016/j.neuron.2009.08.037 19874793 PMC2771122

[B55] Mizuseki K, Royer S, Diba K, Buzsáki G (2012) Activity dynamics and behavioral correlates of CA3 and CA1 hippocampal pyramidal neurons. Hippocampus 22:1659–1680. 10.1002/hipo.22002 22367959 PMC3718552

[B56] O'Keefe J, Recce ML (1993) Phase relationship between hippocampal place units and the EEG theta rhythm. Hippocampus 3:317–330. 10.1002/hipo.450030307 8353611

[B57] Oliva A, Fernández-Ruiz A, Buzsáki G, Berényi A (2016) Spatial coding and physiological properties of hippocampal neurons in the Cornu Ammonis subregions. Hippocampus 26:1593–1607. 10.1002/hipo.22659 27650887

[B58] Orr G, Rao G, Houston FP, McNaughton BL, Barnes CA (2001) Hippocampal synaptic plasticity is modulated by theta rhythm in the fascia dentata of adult and aged freely behaving rats. Hippocampus 11:647–654. 10.1002/hipo.1079 11811658

[B59] Pavlides C, Greenstein YJ, Grudman M, Winson J (1988) Long-term potentiation in the dentate gyrus is induced preferentially on the positive phase of theta-rhythm. Brain Res 439:383–387. 3359196 10.1016/0006-8993(88)91499-0

[B60] Paxinos G, Watson C (1998) The rat brain in stereotaxic coordinates, Ed 4 San Diego, CA: Academic Press.

[B61] Ranck JB (1973) Studies of single neurons in dorsal hippocampal formation and septum in unrestrained rats. Part I. Behavioral correlates and firing repertoires. Exp Neurol 41:461–531. 4355646 10.1016/0014-4886(73)90290-2

[B62] Remy S, Spruston N (2007) Dendritic spikes induce single-burst long-term potentiation. Proc Natl Acad Sci USA 104:17192–17197. 10.1073/pnas.0707919104 17940015 PMC2040482

[B63] Robinson J, Manseau F, Ducharme G, Amilhon B, Vigneault E, El Mestikawy S, Williams S (2016) Optogenetic activation of Septal glutamatergic neurons drive hippocampal theta rhythms. J Neurosci 36:3016–3023. 10.1523/JNEUROSCI.2141-15.2016 26961955 PMC6601761

[B64] Roth L, Leung LS (1995) Difference in LTP at the basal and apical dendrites of CA1 pyramidal neurons in urethane-anesthetized rats. Brain Res 694:40–48. 8974662 10.1016/0006-8993(95)00767-k

[B65] Rudell AP, Fox SE, Ranck JB (1980) Hippocampal excitability phase-locked to the theta rhythm in walking rats. Exp Neurol 68:87–96. 7363990 10.1016/0014-4886(80)90068-0

[B66] Schall KP, Kerber J, Dickson CT (2008) Rhythmic constraints on hippocampal processing: state and phase-related fluctuations of synaptic excitability during theta and the slow oscillation. J Neurophysiol 99:888–899. 10.1152/jn.00915.2007 18046004

[B67] Sejnowski TJ, Paulsen O (2006) Network oscillations: emerging computational principles. J Neurosci 26:1673–1676. 10.1523/JNEUROSCI.3737-05d.2006 16467514 PMC2915831

[B68] Sheremet A, Burke SN, Maurer AP (2016) Movement enhances the nonlinearity of hippocampal theta. J Neurosci 36:4218–4230. 10.1523/JNEUROSCI.3564-15.2016 27076421 PMC4829647

[B69] Shinohara Y, Hosoya A, Yahagi K, Ferecskó AS, Yaguchi K, Sík A, Itakura M, Takahashi M, Hirase H (2012) Hippocampal CA3 and CA2 have distinct bilateral innervation patterns to CA1 in rodents. Eur J Neurosci 35:702–710. 10.1111/j.1460-9568.2012.07993.x 22339771

[B70] Stewart M, Fox SE (1990) Do septal neurons pace the hippocampal theta rhythm? Trends Neurosci 13:163–168. 1693232 10.1016/0166-2236(90)90040-h

[B71] Stumpf C (1965) Drug action on the electrical activity of the hippocampus. Int Rev Neurobiol 8:77–138. 4954552 10.1016/s0074-7742(08)60756-4

[B72] Tesche CD, Karhu J (2000) Theta oscillations index human hippocampal activation during a working memory task. Proc Natl Acad Sci USA 97:919–924. 10639180 10.1073/pnas.97.2.919 PMC15431

[B73] Tort AB, Komorowski RW, Manns JR, Kopell NJ, Eichenbaum H (2009) Theta-gamma coupling increases during the learning of item-context associations. Proc Natl Acad Sci USA 106:20942–20947. 10.1073/pnas.0911331106 19934062 PMC2791641

[B74] Townsend G, Peloquin P, Kloosterman F, Leung LS (2002) Recording and with through silicon multichannel electrodes. Brain Res Protoc 9:122–129. 10.1016/S1385-299X(02)00139-3 12034331

[B75] Vandecasteele M, Varga V, Berényi A, Papp E, Barthó P, Venance L, Freund TF, Buzsáki G (2014) Optogenetic activation of septal cholinergic neurons suppresses sharp wave ripples and enhances theta oscillations in the hippocampus. Proc Natl Acad Sci USA 111:13535–13540. 10.1073/pnas.1411233111 25197052 PMC4169920

[B76] Vanderwolf CH (1969) Hippocampal electrical activity and voluntary movement in the rat. Electroencephalogr Clin Neurophysiol 26:407–418. 4183562 10.1016/0013-4694(69)90092-3

[B80] Winson J (1974) Patterns of hippocampal theta rhythm in the freely moving rat. Electroenceph. Clin. Neurophysiol 36:291–301. 10.1016/0013-4694(74)90171-0 4130608

[B77] Winson J (1978) Loss of hippocampal theta rhythm results in spatial memory deficit in the rat. Science 201:160–163. 663646 10.1126/science.663646

[B78] Wyble BP, Linster C, Hasselmo ME (2000) Size of CA1-evoked synaptic potentials is related to theta rhythm phase in rat hippocampus. J Neurophysiol 83:2138– 2144. 10.1152/jn.2000.83.4.2138 10758123

[B79] Ylinen A, Soltész I, Bragin A, Penttonen M, Sik A, Buzsáki G (1995) Intracellular correlates of hippocampal theta rhythm in identified pyramidal cells, granule cells, and basket cells. Hippocampus 5:78–90. 10.1002/hipo.450050110 7787949

