# Supplementary material for: Long-Term Potentiation and Excitability in the Hippocampus Are Modulated Differently by θ Rhythm
Source: eNeuro. 2018 Nov 22;5(6):ENEURO.0236-18.2018. doi: 10.1523/ENEURO.0236-18.2018 (PMC6325566; doi:10.1523/ENEURO.0236-18.2018)
Supplement: Supplementary Table 1-1 — Parameters (mean ± SE) of curve fit by a single sine wave of the phase-averaged normalized LTP (y) versus theta phase (x) for A. basal and B. apical dendritic excitation. LTP fitted with single sinusoid, either first harmonic y = [A0 + A1 sin (π* (x + A2) /180], or second harmonic y = [B0 + B1 sin (π* (x + B2) /90]. Download Table 1-1, DOC file [file sup_enu-eN-CFN-0236-18-s02.doc]

**Extended Data**

**Table 1-1.** Parameters (mean ± SE) of curve fit by a single sine wave of the phase-averaged normalized LTP (y) versus theta phase (x) for **A.** basal and **B.** apical dendritic excitation. LTP fitted with single sinusoid, either first harmonic y = [A0 + A1 sin (π* (x + A2) /180], or second harmonic y = [B0 + B1 sin (π* (x + B2) /90].

** P<0.005, *P<0.05, R^2^ significantly different from random pairing of x and y values.

# P<0.001, significantly different between basal and apical parameters, unpaired t-test.

**A.**

| Parameter | LTP1 | LTP2 | LTP3 | LTP4 |
| --- | --- | --- | --- | --- |
| 1^st^ harmonic | | | | |
| A0 | 1.086 ± 0.005 | 1.112 ± 0.008 | 1.115 ± 0.015 | 1.106 ± 0.020 |
| A1 | 0.025 ± 0.006 | -0.041 ± 0.010 | -0.033 ± 0.019 | -0.005 ± 0.031 |
| A2 | 88.86 ± 16.87^o^ | -86.11 ± 16.48^o^ | -75.92 ± 39.71^o^ | 26 ± 313^o^ |
| R^2^ | 0.408* | 0.431* | 0.122 | 0.001 |
| 2^nd^ harmonic | | | | |
| B0 | 1.083 ± 0.006 | 1.102 ± 0.010 | 1.097 ± 0.013 | 1.084 ± 0.015 |
| B1 | 0.0078 ± 0.0085 | 0.0326 ± 0.0151 | 0.077 ± 0.020 | 0.107 ± 0.023 |
| B2 | 36.38 ± 35.63^o^ | 62.54 ± 10.33^o^ | 63.13 ± 5.72^o^ | 65.62 ± 4.83^o^ |
| R^2^ | 0.039 | 0.175 | 0.407* | 0.485** |

**B.**

| Parameter | LTP1 | LTP2 | LTP3 | LTP4 |
| --- | --- | --- | --- | --- |
| 1^st^ harmonic | | | | |
| A0 | 1.066 ± 0.009 | 1.02 ± 0.012 ^#^ | 1.032 ± 0.013 ^#^ | 1.037 ± 0.059 |
| A1 | -0.020 ± 0.014 | 0.022 ± 0.020 | -0.018 ± 0.022 | 0.059 ± 0.035 |
| A2 | 49.19 ± 36.88 ^o^ | -4.17 ± 39.81^o^ | 25.94 ± 52.91^o^ | 18.7 ± 25.38^o^ |
| R^2^ | 0.10 | 0.055 | 0.033 | 0.119 |
| 2^nd^ harmonic | | | | |
| B0 | 1.066 ± 0.009 | 1.01 ± 0.007 ^#^ | 1.027 ± 0.010 ^#^ | 1.026 ± 0.009 ^#^ |
| B1 | 0.032 ± 0.011 | 0.0654 ± 0.009 | 0.064 ± 0.012 | 0.132 ± 0.012 |
| B2 | 8.43 ± 10.78^o^ | 22.62 ± 4.38^o #^ | 18.99 ± 5.92^o #^ | 19.25 ± 2.81^o #^ |
| R^2^ | 0.27* | 0.715** | 0.575** | 0.858** |
